# Mechanistic study of leukopenia treatment by Qijiao shengbai Capsule via the Bcl2/Bax/CASAPSE3 pathway

**DOI:** 10.3389/fphar.2024.1451553

**Published:** 2024-09-04

**Authors:** Siyue Jiang, Pengjiao Wang, Xiaodong Sun, Min Zhang, Shuo Zhang, Yu Cao, Yuben Wang, Li Liu, Xiuli Gao

**Affiliations:** ^1^ State Key Laboratory of Functions and Applications of Medicinal Plants and School of Pharmacy, Guizhou Medical University, Guiyang, China; ^2^ Center of Microbiology and Biochemical Pharmaceutical Engineering, Guizhou Medical University, Guiyang, China; ^3^ Experimental Animal Center of Guizhou Medical University, Guiyang, China

**Keywords:** Qijiao Shengbai Capsule, Leukopenia, mitochondrial apoptosis, multi-omics, apoptosis

## Abstract

**Background:**

Leukopenia can be caused by chemotherapy, which suppresses bone marrow function and can impact the effectiveness of cancer treatment. Qijiao Shengbai Capsule (QJSB) is commonly used to treat leukopenia, but the specific bioactive components and mechanisms of action are not well understood.

**Objectives and results:**

This study aimed to analyze the active ingredients of QJSB and its potential targets for treating leukopenia using network pharmacology and molecular docking. Through a combination of serum pharmacochemistry, multi-omics, network pharmacology, and validation experiments in a murine leukopenia model, the researchers sought to understand how QJSB improves leukopenia. The study identified 16 key components of QJSB that act *in vivo* to increase the number of white blood cells in leukopenic mice. Multi-omics analysis and network pharmacology revealed that the PI3K-Akt and MAPK signaling pathways are important in the treatment of leukopenia with QJSB. Five specific targets (JUN, FOS, BCl-2, CASPAS-3) were identified as key targets.

**Conclusion:**

Validation experiments confirmed that QJSB regulates genes related to cell growth and inhibits apoptosis, suggesting that apoptosis may play a crucial role in leukopenia development and that QJSB may improve immune function by regulating apoptotic proteins and increasing CD4^+^ T cell count in leukopenic mice.

## Graphical Abstract

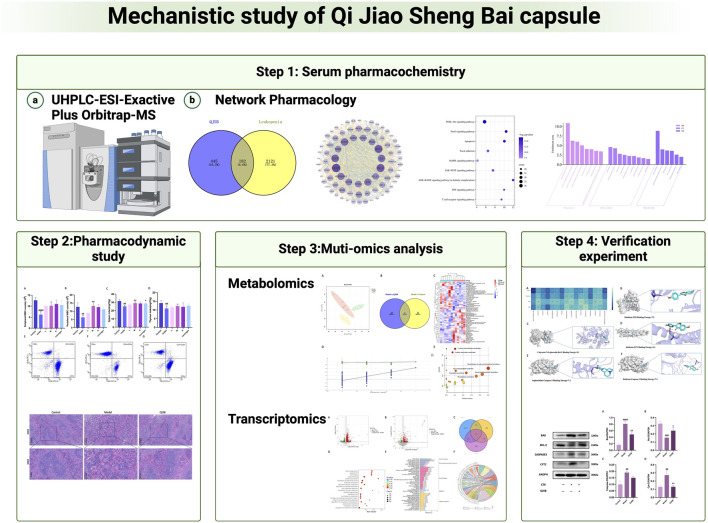

## 1 Introduction

Leukocytopenia is a common adverse reaction associated with abnormalities in the myeloid hematopoietic system, particularly during cancer-related radiation therapy (Shi et al., 2020). The World Health Organization has observed an increasing prevalence of cancer, which is now the leading cause of death globally. Radiotherapy is a common treatment for cancer, but it can lead to various side effects, including leukopenia (Bargetzi, 2006). Patients with leukopenia have a lower number of white blood cells in their bloodstream, which can be caused by different factors such as viral infections, genetic predisposition, and medication side effects (Christen et al., 2017). These patients are at a higher risk of developing infections, particularly hospital-acquired infections and opportunistic pathogens. Cancer is the second leading cause of death worldwide, and chemotherapy remains the primary treatment for killing rapidly dividing cancer cells (Sultana et al., 2014). While chemotherapy is designed to target tumor cells, it can also affect normal cells, particularly bone marrow and white blood cells, leading to a decrease in white blood cell count known as chemotherapy-induced leukopenia. Recent research has shown that chemotherapy can stimulate the immune response against tumor cells, which is essential for successful tumor eradication. Therefore, patients with leukopenia not only face an increased risk of infections but may also experience suboptimal outcomes during chemotherapy (Hodge et al., 2013). It is important for these patients to receive medications that promote an increase in white blood cell count to enhance their immune response and protect against tumor cell destruction. A variety of medications are available for the treatment of leukopenia, including Chinese herbal drugs, Western medicines, biologic hormones, like G-CSF (granulocyte colony-stimulating factor) medications and biological hormones have the potential to significantly raise treatment costs and harmful effects while also causing side effects (Feferman et al., 2017). Chinese medicine is extensively used in the management of leukopenia in our clinic due to its small side effects and also because of its low toxicity and high efficiency (Xu et al., 2011).Leukopenia can be treated with a variety of medications, including Chinese herbal drugs, Western medicines, and biologic hormones like G-CSF. However, biologic hormones can be costly and have harmful side effects. In our clinic, we often use Chinese medicine for treating leukopenia due to its effectiveness and minimal side effects.

The spleen is the largest peripheral immune organ in the body and lymphocytes, macrophages and dendritic cells are the major cellular components of the spleen, of which, up to 85% are lymphocytes (Kaur et al., 1974). Among the various alterations, loss of lymphocytes is the main cause of immunosuppression (Hotchkiss et al., 1999; Hotchkiss et al., 2003; Hotchkiss and Nicholson, 2006), and T cells maintain the body’s immune homeostasis through both cellular and humoral immune responses (Brady et al., 2020). CD4^+^ T cells and CD8^+^ T cells are the most important T cell subpopulations, and indeed, it has been shown that under immunosuppressive conditions, the number of CD4^+^ T cells and CD8^+^ T cells is significantly reduced in mice, leading to a reduced ability to recognize antigens, produce pro-inflammatory cytokines and clear pathogens (Boomer et al., 2011; Condotta et al., 2015; Francois et al., 2018). Apoptosis is known to play an important role in physiology from embryonic implantation and development to adult tissue turnover and T cell selection during T cell maturation (Kerr et al., 1972; Alison and Sarraf, 1992; Cohen et al., 1992). Apoptosis can be induced by two main pathways. The extrinsic pathway is triggered by binding of inducers to cell surface receptors, leading to subsequent caspase-8 activation. The intrinsic “mitochondrial” pathway is driven by disruption of the delicate balance between pro- and anti-apoptotic gene products of the BCL2 superfamily (B cell lymphoma 2) and stress-induced injurious stimuli (van Loo et al., 2001; Madesh et al., 2002). Apoptosis in the mitochondrial pathway is usually activated by cellular stress or injury and is regulated by the interaction of BCL2 family and Caspase family members in mitochondria (Bock and Tait, 2020). It has been reported that the mechanism of reduced T cell numbers and their dysfunction is associated with apoptosis mediated by the mitochondrial pathway, in which CASPASE3 and CASPASE9 are the key enzymes (Venet and Monneret, 2018; Torres et al., 2022). Considering the major link between injury-induced lymphocyte apoptosis and deleterious clinical outcomes, many therapeutic strategies aimed at restoring normal lymphocyte populations and reducing apoptosis in patients have been evaluated in animal models and *in vitro* in patient cells. There is a wide variety of medications available for the treatment of leukopenia, including traditional Chinese medicines, Western medicines, and biologic hormones, such as G-CSF (granulocyte colony-stimulating factor) medications and biologic hormones, which have the potential to significantly increase the cost of treatment and harmful effects, as well as produce side effects (Feferman et al., 2017). Traditional Chinese medicine (TCM) is widely used in the treatment of leukopenia due to its low side effects, low toxicity, and efficacy (Xu et al., 2011).

One specific Chinese medicine called Qijiao Shengbai Capsule (QJSB) has been selected for a national scientific project (Ma et al., 2022). It is a capsule made from a combination of *Indigofera stachyoides* Lindl. (IS, Xue Renshen), *Astragalus membranaceus* (Fisch.) Bge. var. *mongholicus* (Bge.) Hsiao (AM, Huang Qi), *Equus asinum* L. (EA, EJiao), *Angelica sinensis* (Oliv.) Diels (AS, Dang Gui), *Ziziphus jujuba* Mill. (ZJ, Da Zao), *Sophora favescens* Ait. (SF, Ku Shen) and *Epimedium brevicornu* Maxim. (EB, Yin Yanghuo). This formulation has been found to be effective in treating leukopenia (Ma et al., 2022) http://www.theplantlist.org. QJSB contains active ingredients like ferulic acid, Astragalus, and quercetin, which have been shown to improve white blood cell levels and protect cells from damage. QJSB is a complex formulation containing a variety of active ingredients that have been shown to have biological effects (Ma et al., 2011). For example, ferulic acid has been found to restore haematopoietic cells and increase G-CSF levels in mice exposed to whole-body radiation. Astragalus and its total flavonoids have also been shown to enhance the recovery of white blood cell levels in mice with leukopenia (Huang et al., 2013). Additionally, quercetin has been shown to protect endothelial progenitor cells from oxidative damage by inducing autophagy through extracellular signal-regulated kinases (Zhi et al., 2016), while icariin has been found to improve haematopoiesis in mice with myelosuppression induced by cyclophosphamide (Sun et al., 2018). These findings suggest that the various components of QJSB may work together to effectively treat leukopenia, highlighting the complex mechanisms at play.

This study initially analyzed the chemical composition of QJSB and the components that enter the bloodstream using UPLC-Q-Exactive. The potential targets and mechanisms of QJSB in treating leukopenia were then investigated using cyberpharmacology. Pharmacodynamic experiments confirmed the effectiveness of QJSB in improving immunity and treating leukopenia. Transcriptomics identified differentially expressed genes and related signaling pathways in leukopenic mice, while metabolomics analyzed the metabolites and mechanisms of QJSB in treating leukopenia. By integrating cyberpharmacology, transcriptomics, and metabolomics analyses, key targets and signaling pathways of QJSB in managing leukopenia were identified and verified through protein blotting analysis. This study provides insights into the mechanisms of QJSB in treating leukopenia and lays the foundation for further research on its clinical application.

## 2 Materials and methods

### 2.1 Reagents and materials

QJSB samples (Lot Number: 2522012) received from Guizhou Hanfang Pharmaceutical Co., Ltd. (Guiyang, China). CTX (HJ20160467) for injectable was purchased from Jiangsu Hengrui Pharmaceutical Co., Ltd. (Jiangsu, China). Antibodies for Western blotting was purchased from different suppliers. Anti-BCL2 (68103-1-lg), Anti-BAX (660267-1-lg), Anti-CASPASE3 (66470-2-lg), and Anti-CYTC (66264-1-lg) antibodies were purchased from Proteintech, tandem mas labeling (TMT) kits were provided by Thermo 88 Fisher Scientific. RPMI-1640 (HyClone, Thermo Scientific Co, Waltham, MA, United States), red blood cell (RBC) lysing solution (Jimei Biotech, Beijing, China), Anti-CD3e (PerCP-Cy5.5 Hamster), Anti-CD4 (FITC Rat Anti-Mouse), Anti-CD8a (PE Rat Anti-Mouse). All reagents and UPLC-grade reagents were not among the 90 compounds that were examined (formic acid, 89 acetonitrile and methanol, purchased from Merck).

### 2.2 Animal experiments design

#### 2.2.1 Animals

All ICR male mouse [18 ± 22 g, License number: SCXK 127 (Jing) 2019-0010] were obtain from the Animal Ethics Committee of Guizhou Medical University (Guiyang, China). The experiments were performed in a standard mouse laboratory with a 12/12 h light/dark cycle, a temperature of 25 ± 1°C and a humidity of 60% ± 5%. The Animal Ethics Committee of Guizhou Medical University authorized all experiments, and all animals were handled in accordance with the Guidelines for the Care and Use of Animals (License number: SYXK (Qian) 2018-0001; No.2303443).

Eighty mice with temperature fluctuations of less than 0.5°C were chosen for the experiment after a week of acclimatization. Out of these, 60 mice were randomly selected and divided into five groups: normal group, model group, QJSB group (low, medium, high doses), and Leucogen group. For the first 3 days, the normal group received intraperitoneal injections of 0.9% saline, while the other groups were injected with 100 mg/kg of CTX. Over the following 14 days, the normal and model groups were orally administered distilled water following the dosages used in the QJSB group, the QJSB group received low, medium, and high dosages (0.5, 1, and 2 g/kg/d), and the Leucogen group was given risperidone tablets (20 mg/kg/d). The remaining 20 mice were divided into two groups: the QJSB group (four times the human dose of QJSB administered orally daily for three consecutive days) and the normal group (same dose of saline daily for three consecutive days). Blood samples were collected from the eye at 0.5, 1, 1.5, 2, 3, and 4 h after the final administration and stored at −80°C.

#### 2.2.2 Sample preparation and component identification

On the fourth day, 50 *µ*L of blood was randomly drawn from the orbital sinus of six mice in each group, and leukocytes were sorted and counted using an IDXX hematology analyzer. Then, on day 18 of the experiment, blood was withdrawn from the ocular venous plexus and processed. A portion of the spleen tissue was taken for H&E staining and analysis, while the remaining spleen tissue was stored at −80°C. Blood was collected from each group of six mice on the 18th day of the experiment (Cao et al., 2023).

The powder in QJSB was measured at 100 mg and mixed with 1,000 *µ*L of an 80% methanol solution along with grinding beads. The mixture was ground for 5 min and then vortexed for an additional 10 min. After centrifugation at 4°C for 10 min at 12,000 r/min, the upper supernatant was filtered and injected into the UHPLC-ESI-Q-Exactive Plus Orbitrap MS for detection (Tang et al., 2023). For the plasma samples, 1 mL of blank plasma was combined with 1 mL of plasma from the QJSB dose group. The mixture was vortexed with 2 mL of acetonitrile and 2 mL of methanol in an ice bath for 10 min. After centrifugation at 12,000 rpm and 4°C for 10 min, the supernatant was dried under N_2_ and dissolved with 1 mL of methanol. This process was repeated until the residue was dissolved in 200 *µ*L of methanol. After centrifugation at 4°C and 15,000 rpm for 10 min, the supernatant was vortexed for 2 min and then extracted for assay.

The derivatives were separated using a Thermo Vanquish Horizon ultra-performance liquid chromatograph (UPLC) coupled with a Hypersil Gold C_18_ column (2.1 × 100 mm^2^, 1.9 *μ*m) in order to make a preliminary identification of possible active constituents of QJSB extracts. The optimized gradient consisted of phase A (0.1% formaldehyde aqueous solution) and phase B (0.1% formaldehyde acetonitrile solution) and was as follows: Injection volume, flow velocity, and column temperature were 2 μL, 0.3 mL/min, and 40°C, respectively. Samples were measured in both the anionic and the positive ion modes. These were the mass spectrometry statistics. Spray voltage: 3.5/2.5 kV (+/−); capillary temperature: 320°C; vaporiser temperature: 350°C; fullms-ddms normal approach; scanning range: 100–1,500 m/z; resolution: 70,000 (MS1); resolution: 17,500 MS/MS; normalised collision energies (NCEs): 20, 40, and 60th order. Importing raw data into Compound Discoverer The relative mass bias was set to less than 5 ppm when the raw data were imported into Compound Discoverer 3.2 Accurate relative mass and primary and secondary mass spectral fragmentation information were the basis for the analyses, which were performed in Xcalibur software and compared to the m/z Cloud, m/z Vault, Masslist, MoNA, and ChemSpider databases, in addition to the literature. The results were more reliable thanks to the automated identification method mzLogic, and the fragmentation-assisted extrapolation and confirmation of the active ingredient of QJSB were carried out using Mass Frontier 7.0 software (Wang et al., 2023).

### 2.3 Network pharmacology

The active compounds are the result of the analysis of the blood entry components of QJSB and their structures were collected from the PubChem database (https://pubchem.ncbi.nlm.nih.gov/). The obtained compound structure was identified as potential targets for QJSB active ingredients through searching SwissTargetPrediction (www.swisstargetprediction.ch/), The Encyclopedia of Traditional Chinese Medicine database (www.tcmip.cn/ETCM/index.php/Home/Index/index.html), and the TCMSP (the Traditional Chinese Medicine Systems Pharmacology database and analysis platform) (https://tcmspw.com/tcmsp.php). Leukopenia related targets were searching from GeneCards (https://www.genecards.org/), OMIM (Online Mendelian Inheritance in Man) (https://omim.org/), and DrugBank database (https://drugbank.com/) using “Leukopenia” as the keyword, setting the species type to “*Homo sapiens*”, obtaining the target name and remove duplicates, and establish a disease target library. The active compound targets and disease targets were intersected yields a common target for Leukopenia and active compounds. The common targets were imported to STRING (https://cn.string-db.org/) online platform for analysis, and the results are imported to Cytoscape 3.9.1 platform for network Topology process analysis and core targets screening. Key targets were annotated using the DAVID platform for GO function and KEGG enrichment analysis (*P* < 0.01), and the species type was limited to “*Homo sapiens*”, and the results were visualized. “Compound-target-pathway” relationship network diagram of Leukopenia and the active compounds was obtained through using the Cytoscape 3.9.1 software platform, where nodes represented compounds, targets, and pathways, and represented the interaction between them.

### 2.4 Histopathology analysis and CD4^+^, CD8^+^T lymphocyte percentage analysis

The spleen tissues of mice were embedded in paraffin, sliced to a thickness of 4–5 *μ*m, fixed with 4% paraformaldehyde, and examined for any pathological changes. Hematoxylin and eosin (HE) staining was then performed on these spleen sections to further analyze any pathological alterations. The examination of the spleen tissues was done using optical microscopy (Qu et al., 2021).

Six fresh spleens from each group were placed in RPMI-1640 medium with 10% bovine serum. The spleens were crushed using a syringe plunger and a 200-mesh nylon mesh to create a coarse splenic cell suspension. After filtering, the cells were extracted from the interphase and lysed in 2 mL of erythrocyte lysate solution. The lysed cells were then centrifuged at 1,600 rpm for 10 min at 4°C. After another round of centrifugation at the same settings, the cells were lysed again in 2 mL of erythrocyte lysate solution. To stop the lysis process, 4 mL of RPMI-1640 was added to the splenic cell suspension after a 10-min treatment. The mixture was then centrifuged twice using RPMI-1640 for 10 minutes at 1,600 rpm. Following another 10-min centrifugation at 1,600 rpm, PBS was used to adjust the cell count in each group to 1×10^7^ cells/mL. The lymphocyte phenotype was analyzed using flow cytometry. The spleen cells were incubated in the dark for 30 min and then treated with monoclonal antibodies against CD3E (PERCP), CD4 (PE), and CD8A (FITC). The cells were washed with 1.5 mL of PBS, collected, and washed twice with PBS after centrifugation at 1,600 rpm for 10 min at 4°C. They were then resuspended in 500 *µ*L of PBS and analyzed using a BD Biosciences Facscalibur II flow cytometer (United States) (Wang et al., 2020).

### 2.5 Multi-omics study

#### 2.5.1 Metabolomics analysis

The metabolites naturally present in spleen tissue were extracted using organic solvents. To extract the metabolites, a 100 mg sample of spleen tissue was mixed with 0.5 mL of a chilled aqueous solution of methanol and acetonitrile (2:2:1, v/v/v). The sample was homogenized and vortexed for 15 min at a low temperature. Proteins were removed by centrifuging the solution at 15,000 rpm for 15 min and then storing it overnight at −20°C. The resulting supernatant was filtered through a 0.22 *μ*m membrane and placed in a sample vial for metabolomics analysis. The UHPLC-HPI-Q-Exactive Plus Orbitrap-MS system from Agilent Technologies (United States) was used for metabolomics analysis, equipped with a ZORBAX Eclipse Plus C18 column (2.1 × 100 mm^2^, 1.8 *μ*m). The optimal gradient for the analysis included a flow rate of 0.3 mL/min and a series of changes in solvent composition over time. The ion spray voltage, scanning range, and other parameters were set accordingly for the analysis. Data from the mass spectrometry was processed using Compound Discoverer 3.2 software for peak identification and retention time correction. Further analysis was conducted using SIMCA-P 14.1 software for OPLS-DA and PCA analyses. Metabolite identification was confirmed using internal spectral libraries and databases like KEGG and HMDB. MetabolAnalyst 5.0 software was used to analyze changes in plasma metabolites after treatment administration. Metabolic pathways with an influence value greater than 0.1 were considered significant in the analysis (Tang et al., 2023).

#### 2.5.2 Transcriptomic analysis

To conduct transcriptome analysis, spleens from three mice each in the QJSB, model, and control groups were selected. Total RNA was extracted and purified from spleen tissue using Trizol reagent following the manufacturer’s instructions. The concentration and purity of the RNA were determined using Nanodrop 2000, and initial quantification was done using the Qubit 4.0 fluorometer. RNA integrity was tested using agarose gel electrophoresis and the Qsep400 bioanalyzer. The total RNA content needed to be at least 1 *μ*g with a concentration of at least 35 ng to create sequencing libraries with the Illumina TruseqTMRNA Library Preparation Kit. Quality-checked spleen tissue samples were then RNA sequenced using Illumina’s high-throughput sequencing platform (Chen et al., 2023). Data was published on the Michael D. Cloud internet platform. The RNA-seq data were processed by first filtering the Raw Data to obtain clean data with higher quality, and then comparing the data with the corresponding mouse reference genes to obtain the expression amount of each gene. The data were compared with the corresponding mouse reference genes to obtain the expression amount of each gene. The gene expression amounts were used to draw gene expression profiles and the samples were analyzed by bioinformatics. Differential expression analysis was performed using DESeq2 with thresholds of *P-*value ≤ 0.05 and |log2FoldChange| ≥ 0.585 to identify significantly differentially expressed genes (DEGs) across all groups. Functional enrichment analyses using KEGG and GO were conducted to identify DEGs with *P-*value < 0.05 that were enriched in GO terms and metabolic pathways. The metscape platform was used for KEGG pathway analysis and GO functional enrichment. Furthermore, the HMDB database was utilized to explore the relationship between metabolomics and transcriptomics.

### 2.6 Western blot analysis and molecular docking

The spleen tissue was used for total protein extraction following the RIPA lysis buffer protocol from Conviage Biotechnology in China. The proteins were then separated on 10% SDS-PAGE gels and transferred to PVDF membranes. The membrane was blocked with 5% skim milk for 1.5 h at room temperature, and then incubated overnight at 4°C with anti-BCL2 (1:2000), anti-BAX (1:500), anti-CASPASE3 (1:1,000), anti-CYTC (1:500), and anti-GAPDH (1:200) antibodies. After three washes with TBST, the membrane was incubated with HRP rat secondary antibody (1:200) for 1 hour at room temperature. The protein signal was visualized using a gel imager and enhanced chemiluminescence (ECL) detection kit from Proteintech, and analyzed with ImageJ software (Shi et al., 2020).

The crystal structures of two candidate proteins related to leukopenia were obtained from the RCSB protein database. These structures were modified using AutoDock Tool 1.5.7 software, including hydrogenation, dehydration, ligand removal, and amino acid optimization. The modified structures were saved in pdbqt format. The three-dimensional chemical structures of 16 active ingredients were downloaded from PubChem, energy minimized, and saved in MOL.2 format. These compounds were then imported into AutoDock Tool 1.5.7, with all flexible bonds set to be rotatable by default, and saved as docking ligands in pdbqt format. Docking studies were conducted using AutoDock Vina 1.1.2, and the results were visualized with PyMOL.

### 2.7 Data analysis

GraphPad Prism 8.0.2 (San Diego, CA, United States) was used to do statistical analysis on all data, which were represented as mean soil standard deviation (SD). To examine differences between groups, one-way analysis of variance (ANOVA) was utilized; *P* < 0.05 and *P* < 0.01 were deemed statistically significant.

## 3 Results

### 3.1 Characterization of prototypes and metabolites in QJSB

The Xcalibur software was utilized to analyze the data qualitatively and generate an overall ion chromatogram (TIC). By comparing the mass spectrometry data of the samples with relevant literature studies, a total of 95 components from QJSB were tentatively identified (Table 1; Supplementary Figure S1). Based on the initially identified chemical compositions, 16 prototypical components were initially analyzed from the plasma of the administered mice (Table 2; Supplementary Figure S2).

**TABLE 1:** Tentative identification of potential active ingredients in QJSB extracts by UHPLC-ESI-Q-Exactive plus Orbitrap-MS.

no.	RT/min	Name	Molecular Formular	[M+H]^+^	[M+H]^−^	Major fragment ions in positive mode	Major fragment ions in negative mode
1	3.606	Protocatechuic acid-3-0-glc	C_13_H_16_O_9_ (0.25)		315.07278		153.0183 [M-H-Glc]^−^
							109.02826 [M-H-Glc-CO_2_]^−^
2	2.091	Theophylline	C_7_H_8_N_4_O_2_ (3.41)	181.07262		124.03932 [M+H-CH_3_-CH_2_CO]^+^	
3	7.606	2-hydroxy-4-methoxysalicylic acid	C_8_H_8_O_4_ (−4.14)		167.03423		123.04395 [M-H-CO_2_]^−^
4	8.941	7-Hydroxycoumarine	C_9_H_6_O_3_ (−0.37)	163.03926		137.01761 [^1,3^A+]^+^	
5	11.61	3,7-dihydroxy-flavanone	C_15_H_12_O_4_ (0.66)	257.08127		239.06995 [M+H-H_2_O]^+^	
						137.02319 [^1,3^A]^+^	
						119.04922 [^1,3^A-H_2_O]^+^	
6	9.275	Schizandriside	C_25_H_32_O_10_ (2.06)	491.19357			359.15005 [M-H-Xyl]^−^
							341.13968 [M-H-Xyl-H_2_O]^−^
							344.12628 [M-H-Xyl-CH_3_·]^−^
							189.05486 [M-H-Xyl-C_8_H_10_O_4_]^−^
							159.04417 [M-H-Xyl-C_8_H_10_O_4_-CH_2_O]^−^
7	11.558	3,7,4′-trihydroxy-3′-methoxyflavanonol	C_16_H_14_O_6_ (2.2)	303.08698		285.07520 [M+H-H2O]+	
						167.07002 [^1,2^A^+^]^+^	
8	10.509	7,3′,4′,5′-tetrahydroxyflawne	C_15_H_10_O_6_ (−0.39)	287.05496		137.02321 [^1,3^A]^+^	
9	11.353	Daidzein	C_15_H_10_O_4_ (−0.08)	255.06563		237.05363 [M+H-H_2_O]^+^	
						137.02318 [^1,3^A]^+^	
10	13.152	Genistein	C_15_H_10_O_5_ (0.95)	269.04572		225.05446 [M+H-CO-H_2_O]^+^	
						197.05991 [M+H-2CO-H2O]^+^	
11	15.451	Glucopyranose calycosin	C_16_H_12_O_5_ (−2.81)	285.07556		270.05154 [M+H-CH3]^+^	
						181.06444 [^0,3^B]^+^	
						137.02281 [^1,3^A]^+^	
12	11.935	Fisetin	C_15_H_10_O_6_ (−0.62)		285.04080		135.00748 [^1,3^A]^−^
13	14.446	Liquiritigenin	C_15_H_12_O_4_ (−1.06)		255.06590		135.00757 [^1,3^A]^−^
14	12.401	(-)-3,7,3′-trihydroxy-4′-methoxyflavan	C_16_H_16_O_5_ (−1.99)	289.10648		271.09598 [M+H-H_2_O]^+^	
						135.08061 [^1,2^A-H_2_O]^+^	
15	16.908	Farnish	C_16_H_12_O_5_ (−1.93)		283.06113		268.03763 [M-H-CH_3_]^−^
							240.04108 [M-H-CH_3_-CO]^−^
							195.04558 [M-H-CH_3_-OH-2CO]^−^
16	9.042	Coumestrol	C_15_H_8_O_5_ (2.79)	269.04572		251.10614 [M+H-H_2_O]^+^	
17	13.177	4,4′-dihydroxy-2′-methoxychalcone	C_16_H_14_O_4_ (−0.99)		269.08203		253.99792 [M-H-CH_3_]^−^
							237.05627 [M-H-CH_3_OH]^−^
18	12.971	3,7,3′-trihydroxyflavanonol	C_15_H_12_O_5_ (0.05)		271.06110		135.04390 [^1,3^A]^−^
19	11.912	6,4′-dihydroxy-3′-methoxyaurone	C_16_H_12_O_5_ (−2.24)	285.07529		270.05173 [M+H-CH_3_]^+^	
						253.04913 [M+H-CH_3_-OH]^+^	
						225.05444 [M+H-CH_3_-CO-OH]^+^	
20	15.180	3,5,7′-trihydroxyflavanonol	C_15_H_12_O_5_ (0.53)		271.06119		151.03931 [1, 3A]^−^
21	11.379	Genistein	C_15_H_10_O_5_(0.55)		269.04550		239.03494 [M-H-CH2O]^−^
							213.05483 [M-H-CO-2CO]-
22	15.750	7′-hydroxy-3′,4′-dimethoxyisoflavone	C_17_H_14_O_5_ (−0.31)	299.09134		284.06696 [M+H-CH_3_]^+^	
						269.04376 [M+H-2CH_3_]^+^	
						255.06444 [M+H-CO_2_]^+^	
23	12.078	3′-hydroxy-2′,4′-dimethoxy-isoflavan-6-0-glc	C_23_H_28_O_10_ (0.28)		403.16125		301.10815 [M-H-Glc]^−^
							286.08472 [M-H-Glc-CH_3_]^−^
							149.02344 [5A]^−^
24	14.664	Formononetin	C_16_H_12_O_4_ (−1.41)	269.08057		254.05646 [M+H-CH_3_]^+^	
25	15.998	7-hydroxy-4′-methoxyvflavanone	C_16_H_14_O_4_ (0.61)	271.09656		137.05945 [^1,3^A]^+^	
26	24.057	Erycibenin D	C_26_H_30_O_6_ (−1.2)	439.21170		179.03371 [M+H-C6H16O3-C_5_H_16_O_3_]^+^	
27	18.411	Oleanonic acid	C_30_H_46_O_3_ (−1.3)	455.35159		437.34064 [M+H-H_2_O]^+^	
28	1.447	N-methylcytisine	C_12_H_16_N_2_O (1)	205.13374		108.08115 [M+H-C_3_H_9_N-C_3_H_2_]^+^	
						146.06013 [M+H-C_3_H_9_N]_+_	
29	1.535	Cytisine	C_11_H_14_N_2_O (1.61)	191.11818		148.07547 [M+H-C_2_H_4_N]_+_	
30	3.527	Matrine	C_15_H_24_N_2_O(−1.66)	249.19566		150.12787[M+H-H_2_O-CH_2_-C_4_H_5_N]^+^	
						148.11189 [M+H-H_2_O-CH2-C_4_H_5_NH_3_]^+^	
31	4.587	Sophoridine	C_15_H_24_N_2_O (−1.67)	249.19582		150.12735[M+H-H2O-CH_2_-C_4_H_5_N]^+^	
						148.11201 [M+H-H2O-CH_2_-C_4_H_5_N-H_2_]^+^	
32	2.033	Oxymatrine	C_15_H_24_N_2_O_2_ (0.98)	265.19153		247.18013 [M+H-H_2_O]^+^	
33	6.726	9α-hydroxymatrine	C_15_H_24_N_2_O_2_ (2.49)	265.19171		150.12755 [M+H-CO-CH_2_-C_3_H_5_N]^+^	
34	8.891	Sophoranol	C_15_H_24_N_2_O_2_ (2.49)	265.19202		247.1808 [M+H-H_2_O]^+^	
						219.18596 [M+H-H_2_O-CO]^+^	
35	2.997	9α-hydroxy-oxysophocarpine	C_15_H_24_N_2_O_3_ (2.09)	281.18619		150.12753 [M+H-2H_2_O-CO-CH_2_-C_2_HN]^+^	
36	7.878	5α-hydroxysophocarpine	C_15_H_22_N_2_O_2_ (1.79)	263.17587		245.16455[M+H-H_2_O]^+^	
						189.13849 [M+H-H_2_O-CO-H_2_]^+^	
37	1.892	9α-hydroxysophocarpine	C_15_H_22_N_2_O_2_ (1.9)	263.17575		245.16423[M+H-H_2_O]^+^	
						148.11188 [M+H-H_2_O-CO-CH_2_-C_2_HN-H_2_]^+^	
38	8.227	3′-hydroxy-4′-methoxy-isoflavone-7-0-api-0-glc	C_27_H_30_O_14_ (1.7)	579.17267		285.07516 [M+H-132-162]^+^	
						270.05176 [M+H-Xyl-Glc-CH_3_]^+^	
39	10.534	5-hydroxy-4′-methoxy-isoflavone-7-0-glc-0-api	C_27_H_30_O_14_ (1.45)		577.15588		283.06052 [M-H-Xyl-Glc]^−^
							268.03851 [M-H-Xyl-Glc-CH_3_]^−^
40	11.149	7-hydroxy-3′-methoxy-isofavone	C_16_H_12_O_4_ (−0.76)	269.08069		254.05656 [M+H-CH_3_]^+^	
						137.62299 [^1,3^A]^+^	
41	11.935	Kushenol H or Kushenol K	C_26_H_32_O_8_ (−1.49)	473.21713		455.20468 [M+H-H_2_O]^+^	
						179.03362 [^1,3^A-hydroxylationlavandulyl]+	
42	15.897	Kushenol H isomer	C_26_H_32_O_8_ (−1.38)	473.21707		455.35190 [M+H-H2O]^+^	
						437.34036 [M+H-2H_2_O]^+^	
43	17.517	Kurarinol	C_26_H_32_O_7_ (−0.14)	457.22205		179.0335 [^1,3^A-hydroxylationlavandulyl]+	
44	19.090	Kuraridinol	C_26_H_32_O_7_ (−0.14)	455.20749			293.17599 [^1,4^A]^−^
							161.02341 [1, 4B]^−^
45	14.494	2-hydroxy-isxanthohumol	C_21_H_22_O_6_ (−0.44)		369.13397		341.13922 [M-H-CO]^−^
							207.10194 [^1,4^A]^−^
46	18.764	8-isopentenyl-7,4′-dihydroxy-5-methoxy-flavanonol	C_21_H_22_O_6_ (0.02)	371014923		235.09613 [^1,3^A]^+^	
						179.03365 [^1,3^A-isopentenyl]^+^	
47	14.070	Sophorai soflavanone A	C_21_H_22_O_6_ (−0.13)	371.14905		325.14282 [M+H-H_2_O-CO]^+^	
						179.03363 [5A]^+^	
48	21.411	Xanthohumol	C_21_H_22_O_5_ (0.64)	355.15417		299.09042 [M+H-isopentengl]^+^	
						179.03362 [Ⅱa-isopentenyl-CO]^+^	
49	17.149	Kushenol Q	C_25_H_30_O_7_ (−0.94)		441.19189		279.16022 [1, 4A]^−^
50	18.486	Kushenol Q isomer	C_25_H_30_O_7_ (−0.58)		441.19186		279.16006 [1, 4A]^−^
							161.02344 [1, 4B]^−^
51	20.018	Kushenol L	C_25_H_28_O_7_ (0.37)		439.17633		261.14957 [^1,4^A]^−^
							177.01845[^1,4^B]^−^
							149.02336[1, 4B-CO]^−^
52	17.419	Kurarinone	C_26_H_30_O_6_ (−1.28)	439.21155		303.15851 [^1,3^A]^+^	
						179.03363 [^1,3^A-lavandulyl]^+^	
53	20.649	Kurarinone isomer	C_26_H_30_O_6_ (−1.26)	439.21146		421.20728 [M+H-H_2_O]^+^	
						303.15836 [^1,3^A]^+^	
54	17.395	Desmethylxanthohumol	C_20_H_20_O_5_ (−1.13)	339.12344			245.08165 [ⅣA]^−^
55	18.965	Kushenol R	C_26_H_30_O_5_ (0.31)	423.21652		303.15836 [^1,3^A]^+^	
						179.03368 [^1,3^A-lavanduly]^+^	
56	21.765	8-lavandulyl Kaempferol	C_26_H_30_O_5_ (0.26)	423.21716		299.05496 [M+H-lavandulyl]^+^	
57	17.125	Kushend C	C_25_H_26_O_7_ (0.08)	439.17596		315.07663 [M+H-lavandulyl]^+^	
58	19.304	2′-methoxykurarinone	C_27_H_32_O_6_ (−0.55)	453.22684		303.15845 [^1,3^A]^+^	
						179.03362 [^1,3^A-lavandulyl]^+^	
59	19.522	Nor kurarinone or Sophoraflavanone G	C_25_H_28_O_6_ (0.19)		423.18167		313.14383 [5A]^−^
							261.14966 [1, 4A]^−^
60	23.936	Sophoraflavanone G	C_25_H_28_O_6_ (0.13)		423.18146		261.14963 [1, 4A]^−^
							135.04408 [1, 3B]^−^
61	10.784	Dihydrodemethylicaritin-7-0-di-glc	C_32_H_40_O_16_ (−1.07)		679.22363		283.02548 [M-H-2Glc-CO-CO_2_]^−^
62	3.071	Quinic acid	C_7_H_12_O_6_ (−3.44)		191.005539		145.04961 [M-H-H_2_O-CO]^−^
63	7.219	Quinic acid isomer	C_7_H_12_O_6_ (−2.81)		191.05585		173.04482[M-H-H_2_O]^−^
64	7.316	Chlorogenic acid	C_16_H_18_O_9_ (2.11)		353.08844		191.05534 [M-H-C_9_H_6_O_3_]^−^
							135.04408 [M-H-C_7_H_10_O5-CO_2_]^−^
							161.02362 [M-H-C_7_H_10_O_5_-H_2_O]^−^
65	6.366	Neochlorogenic acid	C_16_H_18_O_9_ (1.71)		353.08868		191.05528 [M-H-C_9_H_6_O_3_]^−^
							135.04398 [M-H-C_7_H_10_O_5_-CO_2_]^−^
66	7.948	3-coumaroylquinic acid	C_16_H_18_O_9_ (1.71)		337.09433		191.05531 [M-H-C_9_H_6_O_2_]^−^
							163.03906 [M-H-C_7_H_10_O_5_]^−^
67	10.582	Epimedin B isomer	C_38_H_48_O_19_ (−0.97)		807.27063		645.21857 [M-H-Glc]^−^
							351.08734 [M-H-Glc-Rha-Xyl-CH_3_·-H·]^−^
68	10.336	Ikarisoside C	C_38_H_48_O_20_ (−0.67)		823.26581		661.21368 [M-H-Glc]^−^
							353.10226 [M-H-2Glc-Rha]^−^
69	10.436	Epimedoside E	C_37_H_46_O_19_ (−1.17)		793.25586		631.20343[M-H-Glc]^−^
							353.09479 [M-H-Glc-Xyl-Rha]^−^
70	10.082	Icarisoside B	C_32_H_38_O_15_ (−1.4)		661.21326		515.15601 [M-H-Glc]^−^
							353.10287 [M-H-Glc-Rha]^−^
71	10.835	3′-hydroxyicariine-3-0-rha-7-0-glc	C_39_H_50_O_20_ (−1.93)		837.28131		675.22943 [M-H-Glc]^−^
							383.11316 [M-H-Glc-2Rha]^−^
72	12.032	icariin isomer	C_33_H_40_O_15_ (−1.01)		675.22882		367.11844 [M-H-Glc-Rha]^−^
							352.09445 [M-H-Glc-Rha-CH_3_·]^−^·
73	14.590	icariin	C_33_H_40_O_15_ (−2.06)		675.22870		323.09409 [M-H-Glc-Rha·-CH_3_·-CO-H·]^−^
							311.09293 [M-H-Glc-Rha-C_4_H_8_]^−^
74	15.775	icariin isomer	C_33_H_40_O_15_ (−1.85)		675.22876		367.11835 [M-H-Glc-Rha]^−^
							352.09464 [M-H-Glc-Rha-CH3]^−^
							323.09152[M-H-Glc-Rha-CH_3_·-CHO·]^−^
75	14.349	Sagittatoside B	C_32_H_38_O_14_ (−1.98)		645.21802		351.09009 [M-H-Xyl-Rha-CH3·-H·]^−^
							295.09763 [M-H-Xyl-Rha-CH_3_·-2CO-H·]^−^
76	12.177	Sagittatoside B isomer	C_32_H_38_O_14_ (−0.48)		645.21857		367.11774 [M-H-Xyl-Rha]^−^
							323.09177[M-H-Xyl-Rha-CH_3_·-CHO·]^−^
78	13.943	Demethy lanhydroicaritin-7-0-glc	C_26_H_28_O_11_ (0.32)		515.15564		353.10242 [M-H-Glc]^−^
							297.04117[M-H-Glc-Rha-C_4_H_8_]^−^
79	12.376	2″-0-rhamnoshl icariside Ⅱ	C_33_H_40_O_14_ (−1.25)		659.23370		366.11075 [M-H-2Rha-H·]^−^
							351.08755 [M-H-2Rha-CH3·-H·]^−^
80	16.317	2″-0-Orhamnoshl icariside Ⅱ isomer	C_33_H_40_O_14_ (−1.98)		659.13318		366.11050[M-H-2Rha-H·]^−^
							323.09326[M-H-Xyl-Rha-CH3·-CO-H·]^−^
81	17.003	Baohuoside Ⅰ	C_27_H_30_O_10_ (−0.92)		513.17615		366.11084 [M-H-Rha-H·]^−^
							351.08731 [M-H-Rha-CH_3_·-H·]^−^
82	8.891	Calycosin-7-0-glc	C_22_H_22_O_10_(0.97)	447.17615		285.07520 [M+H-Glc]^+^	
						270.05182 [M+H-Glc-CH_3_]^+^	
83	12.497	Ferulic acid	C_10_H_10_O_4_ (1.1)	195.06540		177.05360 [M+H-H_2_O]^+^	
						117.03253 [M+H-H_2_O-CH_3_OH-CO]^+^	
84	10.460	isomucronulatol-4′-0-api	C_22_H_26_O_9_ (−1.02)	435.16455		417.15341 [M+H-H2O]^+^	
85	11.149	Ononin	C_22_H_22_O_9_ (−1.11)	431.13364		269.08017 [M+H-Glc]^+^	
						254.05681 [M+H-GlcCH_3_]^+^	
86	10.913	Calycosin-7-0-glc(OAc)	C_24_H_24_O_11_ (0.63)	489.13919		285.07516 [M+H-GlcOAc]^+^	
						270.05191 [M+H-GlcOAc-CH_3_]^+^	
87	11.711	Methylinissolin-3-0-glc	C_23_H_26_O_10_ (−0.85)	463.15973		301.10641 [M+H-Glc]^+^	
88	11.711	3-hydroxy-9,10-dimethoxy-pterocarpane	C_17_H_16_O_5_ (−0.3)	301.10712		167.07005 [M+H-C_7_H_6_O_2_]^+^	
89	15.354	7,2′-dinhydroxy-3′,4′-dimethoxyisoflavane	C_17_H_18_O_5_ (−0.16)	303.12265		167.07014 [5A+H_2_O]^+^	
						149.05959 [5A]^+^	
90	12.153	Kumatakenin	C_17_H_14_O_6_ (−0.11)	315.08670		300.06241 [M+H-CH_3_]^+^	
						167.03392 [^1,3^A]^+^	
91	15.002	10-hydroxy-3,9-dimethoxy-pterocarpane isomer	C_17_H_16_O_5_(−0.29)	301.10724		167.07007 [M+H-C_7_H_6_O_2_]^+^	
92	15.377	Senkyunolide or isomer	C_12_H_14_O_3_ (−3.12)		205.08629		161.09618 [M-H-CO_2_]^−^
93	14.664	butylidenephthalide isomer	C_12_H_12_O_2_ (−1.07)		169.09077	171.08009 [M+H-H2O]^+^	
						143.08528 [M+H-H_2_O-CO]^+^	
94	17.759	Ligustillide isomer	C_12_H_14_O_2_ (−0.69)	191.10684		173.09592 [M+H-H_2_O]^+^	
						143.10100 [M+H-H_2_O-CO]^+^	
95	19.450	E-butylidenephthalide	C_12_H_12_O_2_ (−1.05)	191.10658		171.08018 [M+H-H_2_O]^+^	
						153.06973 [M+H-2H_2_O]^+^	
						143.08542 [M+H-H_2_O-CO]^+^	

**TABLE 2:** Tentative Identification of components from mouse plasma in QJSB Extracts by UHPLC-ESI-Q-Exactive Plus Orbitrap-MS.

no.	RT/min	Name	Molecular Formular	[M+H]^+^	[M+H]^−^	Major fragment ions in positive mode	Major fragment ions in negative mode
1	1.438	Nicotinamide	C_6_H_6_N_2_O (1.51)	123.05551+		123.05527	
						96.04474	
						80.05002	
2	6.464	Sophoridine	C_15_H_24_N_2_O (−0.14)	249.1962+		150.12749	
						148.11174	
3	5.808	Matrine	C_15_H_24_N_2_O (−0.14)	249.19621+		150.12744	
						148.11182	
4	22.941	dibutyl phthalate	C_16_H_22_O_4_ (−0.65)	279.15909+		149.01212	
5	15.437	Calycosin-7-0-glc	C_22_H_22_O_10_ (−0.9)	447.12805 +		—	
6	22.838	Palmiticacid	C_16_H_32_O_2_ (−2.67)		255.23253 −	—	
7	11.902	azelaic acid	C_9_H_16_O_4_ (−4.64)		187.09674 −		169.08598
							125.09599
8	10.308	5alpha-Hydroxysophocarpine	C_15_H_22_N_2_O_2_ (0.51)	263.17554+		245.16478	
9	21.892	Linoleicacid	C_18_H_32_O_2_ (−1.35)		279.23260−		279.23252
							162.86351
10	1.558	Citricacid	C_6_H_8_O_7_ (−4.35)		191.01881−		173.00868
							129.01814
11	1.668	n-methylcytisine	C_12_H_16_N_2_O (0.4)	205.13362+		108.08101	
						146.06015	
12	25.337	Stearicacid	C_18_H_36_O_2_ (−1.51)		283.26376−	—	
13	23.368	oleic acid	C_18_H_34_O_2_ (−1.86)		281.24808−		281.24854
14	2.083	Coumarin	C_9_H_6_O_2_ (0.5)	147.04413+		119.04917	
						91.0546	
15	30.333	methyl palmitate	C_17_H_34_O_2_ (0.05)	288.77878+		102.09167	
						71.08611	
16	12.82	Daidzein	C_15_H_10_O_4_ (−0.34)	255.06522+		—	

### 3.2 Screening of potential QJSB targets for treating WBC

**FIGURE 1:**
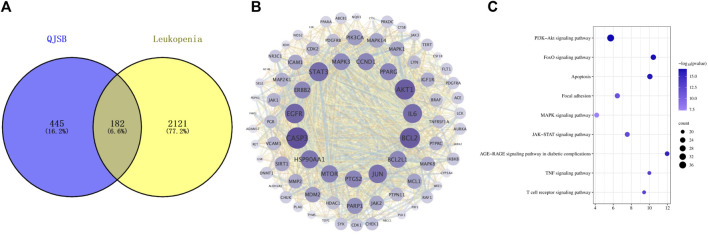
The target information of QJSB in the treatment of leukopenia. **(A)** Venn diagram of component targets-disease targets. **(B)** PPIs. **(C)** Results of KEGG enrichment analysis of the potential targets.

In order to investigate how QJSB works against leukopenia, a network pharmacology analysis was conducted. A total of 670 targets related to 16 active ingredients of QJSB were gathered from SwissTargetPrediction, the Encyclopedia of Traditional Chinese Medicine database, and the TCMSP. Additionally, 2,303 targets associated with leukopenia were collected from GeneCards, OMIM, and DrugBank database. After comparing the two sets of targets, 182 potential targets for QJSB treatment of leukopenia were identified (Figure 1A). These targets were then analyzed using the STRING platform to create PPI diagrams with the help of Cytoscape 3.9.1 software (Figure 1B). The importance of the active ingredients was assessed based on their degree, betweeness, and closeness in the network, with fifty targets scoring above average. Notably, AKT1, EGFR, IL6, STAT3, BCL2, CASP3, and JUN were identified as the top seven targets (degree >80) and may be key targets of QJSB in treating leukopenia. KEGG pathway enrichment analysis was then conducted to predict the potential mechanism of QJSB in treating leukopenia. The KEGG pathway enrichment analysis produced 158 enrichment results with *P-*value < 0.05 (Figure 1C), significantly enriched to PI3K-Akt, apoptosis, and other pathways, which may be the key pathways for QJSB treatment of leukopenia. The composition-target-pathway network illustrates how different targets are associated with different components and how various components are connected to multiple targets. Using Cytoscape 3.7.1, a blood entry component-target-pathway diagram was created (Supplementary Figure S3). The network interaction revealed that each component had more than 5 therapeutic targets, with almost half of the targets being influenced by more than two components. Additionally, all enriched pathways had over 10 therapeutic targets.

### 3.3 Pharmacodynamic verification of QJSB in the treatment of WBC

#### 3.3.1 Effect of QJSB on peripheral leukocytes in leukopenic mice

**FIGURE 2:**
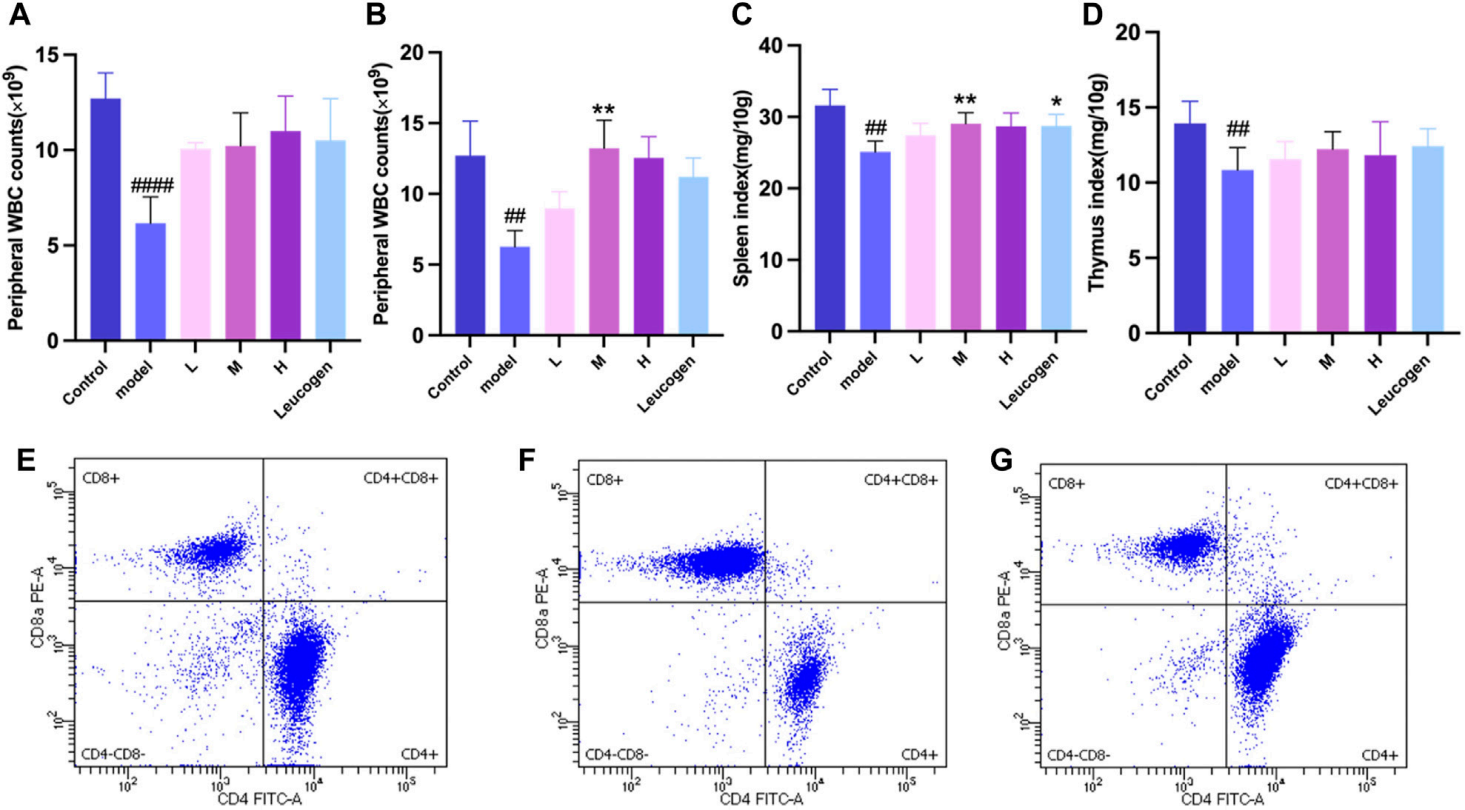
The effect of QJSB on the peripheral WBC **(A)** in leucopenia mice induced by CTX. After 2 weeks of treatment, QJSB increased peripheral WBCs **(B)** The effect of QJSB on the spleen index **(C)** The effect of QJSB on the thymus index **(E)** Data on lymphocyte subpopulations in the spleens of control mice; **(F)** Data on lymphocyte subpopulations in the spleens of Model mice; **(G)** Data on lymphocyte subpopulations in the spleens of QJSB mice. Data are presented as means ± SD. ### Represent *p* < 0.001 vs. normal group and ∗, ∗∗, and ∗∗∗ represent *p* < 0.05, 0.01, and 0.001 vs. model group, respectively.

Peripheral white blood cell counts were considerably lower following cyclophosphamide modeling. Figure 2A illustrates how the QJSB group’s peripheral white blood cell count was considerably higher than that of the model group 1 week after QJSB was administered. Figure 2B illustrates the similar outcomes that were also attained in leukopenia model mice given QJSB for 2 weeks.

#### 3.3.2 Effect of QJSB on organ index in leukopenic mice

Figures 2C, D demonstrates that there was no significant difference (*P* > 0.05) between the QJSB low-dose group L, QJSB medium-dose group M, and QJSB high-dose group H and the Control group with regard to the spleen index and thymus index of leukopenic mice. The QJSB medium dose group clearly impacted the spleen index of leukopenic mice.

#### 3.3.3 CD4^+^, CD8^+^ T lymphocytes percentage analysis QJSB effects on leucopenia disease immune function in mice

As shown in Figures 2E–G, compared with normal group, CD4^+^T lymphocyte percentage and CD4^+^/CD8^+^ ratio of mice in model group were significantly decreased *(P* < 0.05), and CD8^+^T lymphocyte percentage was significantly increased (*P* < 0.05). Compared with model group, CD4^+^T lymphocyte percentage in QJSB group was significantly increased (*P* < 0.05), CD8^+^T lymphocyte percentage was significantly decreased (*P* < 0.05), and CD4^+^/CD8^+^ ratio was significantly increased (*P* < 0.05). Data on mice spleen lymphocyte subpopulations are shown in Table 3.

**TABLE 3:** Effects of QJSB on the percentages of CD4+ and CD8+ lymphocytes in the spleen.

	N	CD4+%	CD8%	CD4+/CD8+
Control	8	65.24 ± 3.13	29.24 ± 3.06	2.3 ± 0.42
Model	8	47.61 ± 12.84	48.84 ± 13.49	1.13 ± 0.4
QJSB	8	65.3 ± 11.19	30.09 ± 10.73	2.65 ± 2.06

#### 3.3.4 Histopathologic analysis QJSB treatment of leucopenia disease in mice spleen histopathological effect

**FIGURE 3:**
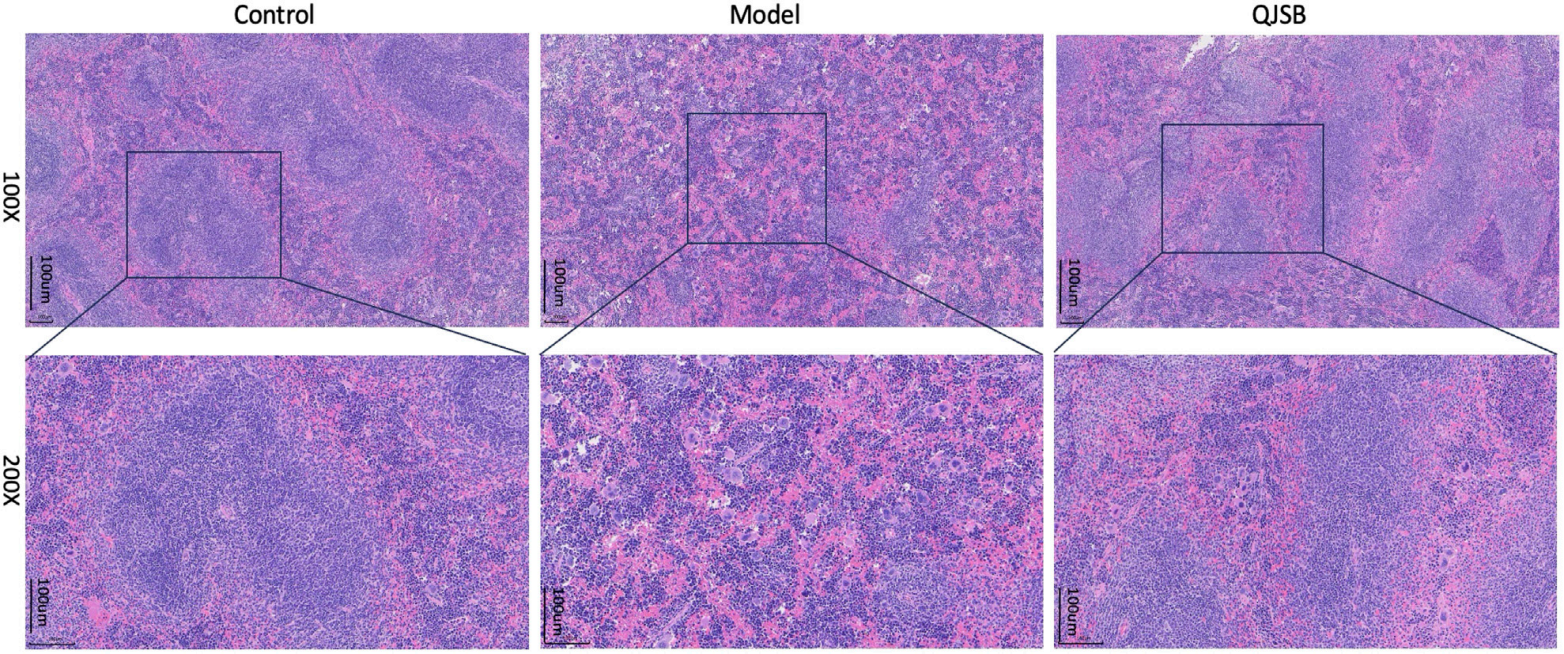
Effect of QJSB on spleen tissue in mice (H&E control, model, QJSB magnification ×100 and ×200).

In the blank group, as shown in Figure 3, the splenic subcapsular parenchyma had a clear structure, the white and red pulp were clearly structured, the white pulp was developed, there were abundant splenic corpuscles, the lymphoid nodules were clearly structured, and the boundaries of nucleus and cytoplasm were clearly defined. Model group: Spleen of mice showed different degree of injury, the distinction between red and white pulp of spleen was not clear, the white pulp was reduced, the structure was disorganized, most of them were scattered in small chunks in the red pulp, and the spleen sinus was congested. QJSB group: The spleen structure of most mice returned to normal, the white pulp structure was clear, the red pulp structure was restored, the hemorrhagic spots were significantly reduced, and the structure of lymph nodules basically returned to normal.

### 3.4 Results of multi-omics studies

#### 3.4.1 Metabolomics analysis of QJSB in treating WBC

**FIGURE 4:**
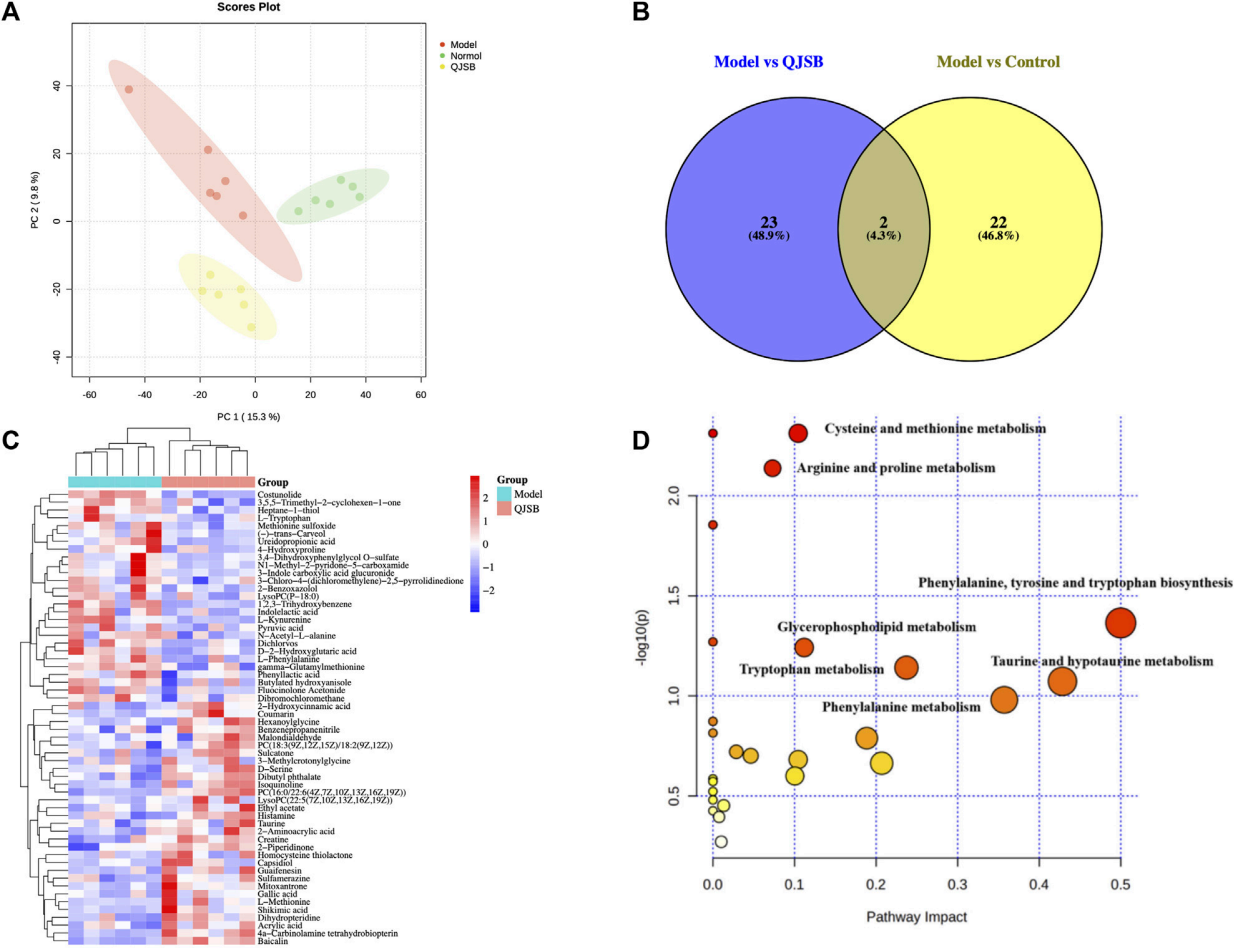
QJSB regulates metabolites in serum of CTX-induced leukopenia mice. **(A)** PLS-DA score plot in ESI ± mode. **(B)** Venn diagram of metabolites **(C)** Clustering heat map of potential differential metabolites in serum. **(D)** Key metabolic pathways of differential metabolites.

Using PCA and PLS-DA in ESI ± mode (positive and negative ion mode), the metabolic profiles of the control group, model group, and QJSB group were distinguished. The serum samples from the QJSB, control, and model groups showed segregation in PCA (Figure 4A). The OPLS-DA analysis further confirmed the reliability and repeatability of the test method by separating the different groups considerably. Metabolite selection and potential assessment were based on VIP >1.5, *p* < 0.05, and FC > 1.2 or <0.833 criteria, leading to the identification of 58 different types of metabolites in the three groups. A comparison between the QJSB group and the model group revealed 29 metabolites with positive regulation and 29 with inverse regulation following QJSB treatment (Figure 4B). A heat map was created to visualize the variations across the metabolites in the three groups (Figure 4C). Metabolic pathway enrichment analysis using MetaboAnalyst 5.0 identified key metabolic pathways, including taurine and hypotaurine metabolism, phenylalanine metabolism, tryptophan metabolism, cysteine and methionine metabolism, arginine and proline metabolism, glycerol phospholipid metabolism, tyrosine and tryptophan biosynthesis, and taurine and hypotaurine metabolism (Figure 4E).

#### 3.4.2 Transcriptomic analysis of QJSB in treating WBC

**FIGURE 5:**
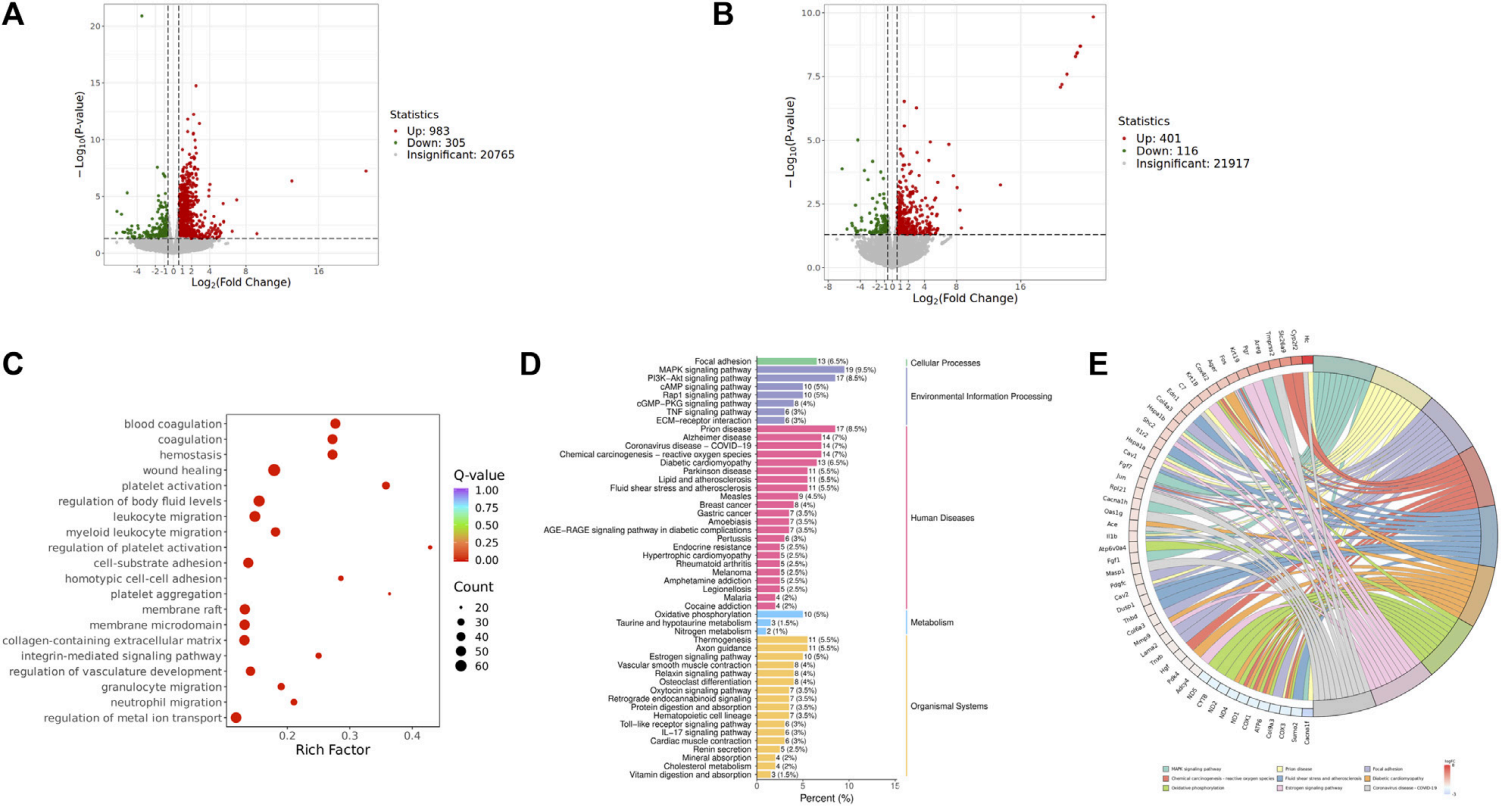
Transcriptomics analysis of QJSB on CTX-induced leukopenia mice. **(A)** Relative volcano plot in Model and Control groups. **(B)** Relative volcano plot in Model and QJSB groups. **(C)** GO enrichment analysis of differently expressed genes. **(D)** KEGG enrichment analysis of differently expressed genes. **(E)** Enriched chord diagram of pathways.

We focused on analyzing the changes in spleen gene expression in the control group, model group, and QJSB group based on the pharmacodynamic results. DESeq2 was used for differential expression analysis with biological replicates. A total of 1,288 differentially expressed genes were identified between the model group and control group, with 983 upregulated and 305 downregulated genes. Additionally, 517 genes were found to be differentially expressed between the model group and QJSB treatment group, including 116 downregulated and 401 upregulated genes (Figures 5A, B). Pathway enrichment analysis revealed that QJSB treatment affected pathways such as focal adhesion, PI3K-Akt, and MAPK signaling pathways (Figure 5E). Furthermore, JUN and FOS were identified as key regulators in multiple pathways related to leukopenia treatment (Figure 5F). GO enrichment analysis showed that QJSB had significant effects on the regulation of epithelial cell proliferation, extracellular matrix organization, and cellular structure in mice with leukopenia induced by CTX (Figure 5D). Interestingly, multiple pathways involved in JUN and FOS may be key genes for QJSB in the treatment of leukopenia.

### 3.5 Molecular docking identified potential pharmaceutical ingredients of QJSB

**FIGURE 6:**
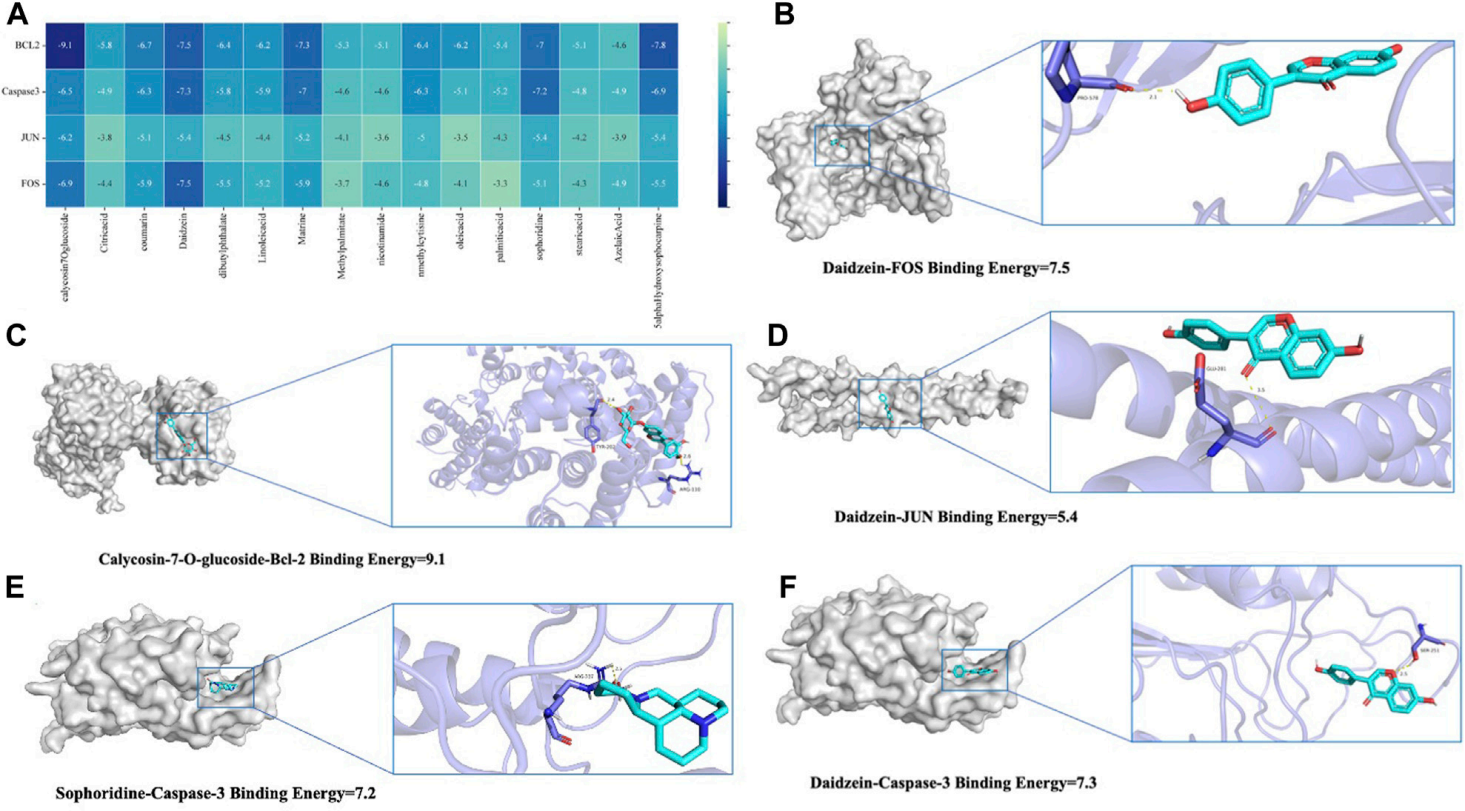
Molecular models of 16 QJSB core active components binding to the four most promising target proteins on leukopenia. **(A)** Heat map of minimum binding energy **(B–F)** Visualization of molecular docking with minimum binding energy less than −7 kJ/mol.

To thoroughly analyze the incoming blood components, we conducted molecular docking of 16 active components with key proteins. The stability of the protein receptor and small molecule ligand binding is determined by binding energy, with lower energy indicating a more stable binding conformation. A binding energy below 0 allows the ligand to freely bind to the receptor, while a value below −5.0 kcal/mol indicates strong binding activity with the target protein. Figure 6A shows the minimum binding energy from molecular docking. Furthermore, partial results of molecular docking are visually represented in Figures 6B–F. The affinity between compounds and targets mostly falls below −5.0 kcal/mol, suggesting a strong potential for close interaction between key compounds and targets. This indicates that QJSB may have therapeutic effects on Leukopenia through these compounds.

### 3.6 Integrated analysis of key biological processes regulated by QJSB

**FIGURE 7:**
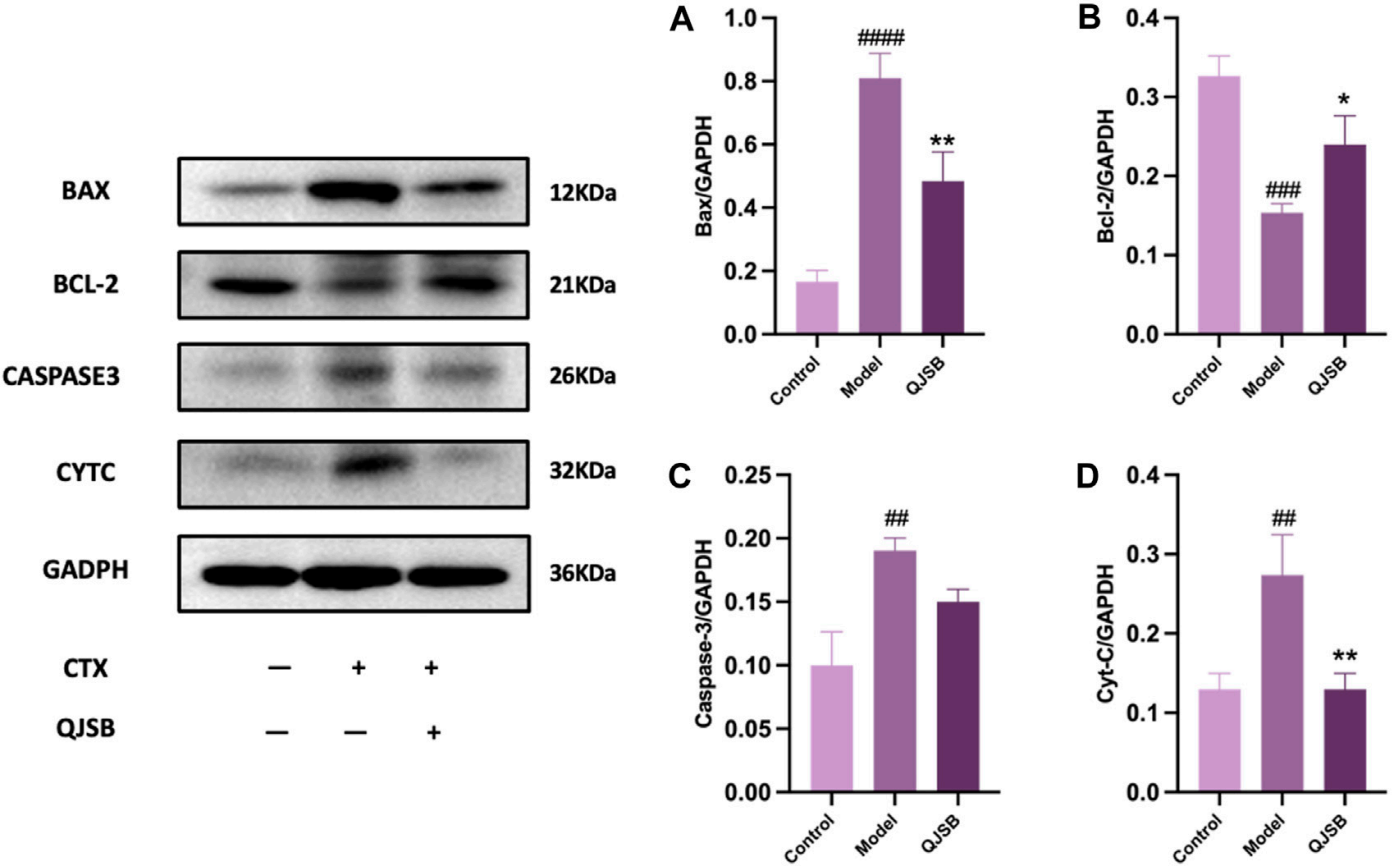
Effect of QJSB on protein expression of apoptosis in spleen tissue of leukopenia. Data are presented as means ± SD. ### Represent *p* < 0.001 vs. normal group and ∗, ∗∗, and ∗∗∗ represent *p* < 0.05, 0.01, and 0.001 vs. model group, respectively. Quantification of Bax, Bcl-2, Caspase-3, Cyt-C protein expression **(A–D)**.

Through molecular docking analysis, it was discovered that QJSB has a significant impact on the expression of apoptotic proteins BAX, BCL2, CASPASE3, and CYTC in mice with leukopenia (Figures 7A–D). Western blot analysis revealed that the Model group of mice had higher levels of CYTC (*P* < 0.05), BAX (*P* < 0.0001), and CASPASE3 (*P* < 0.05) compared to the Control group, while BCL2 expression was lower (*P* < 0.01). Treatment with QJSB led to decreased levels of CYTC (*P* < 0.05), BAX (*P* < 0.05), and CASPASE3 (*P* > 0.05) compared to the Model group, with an increase in BCL2 expression (*P* < 0.05), indicating that QJSB effectively inhibited spleen cell apoptosis in CTX mice.

**FIGURE 8:**
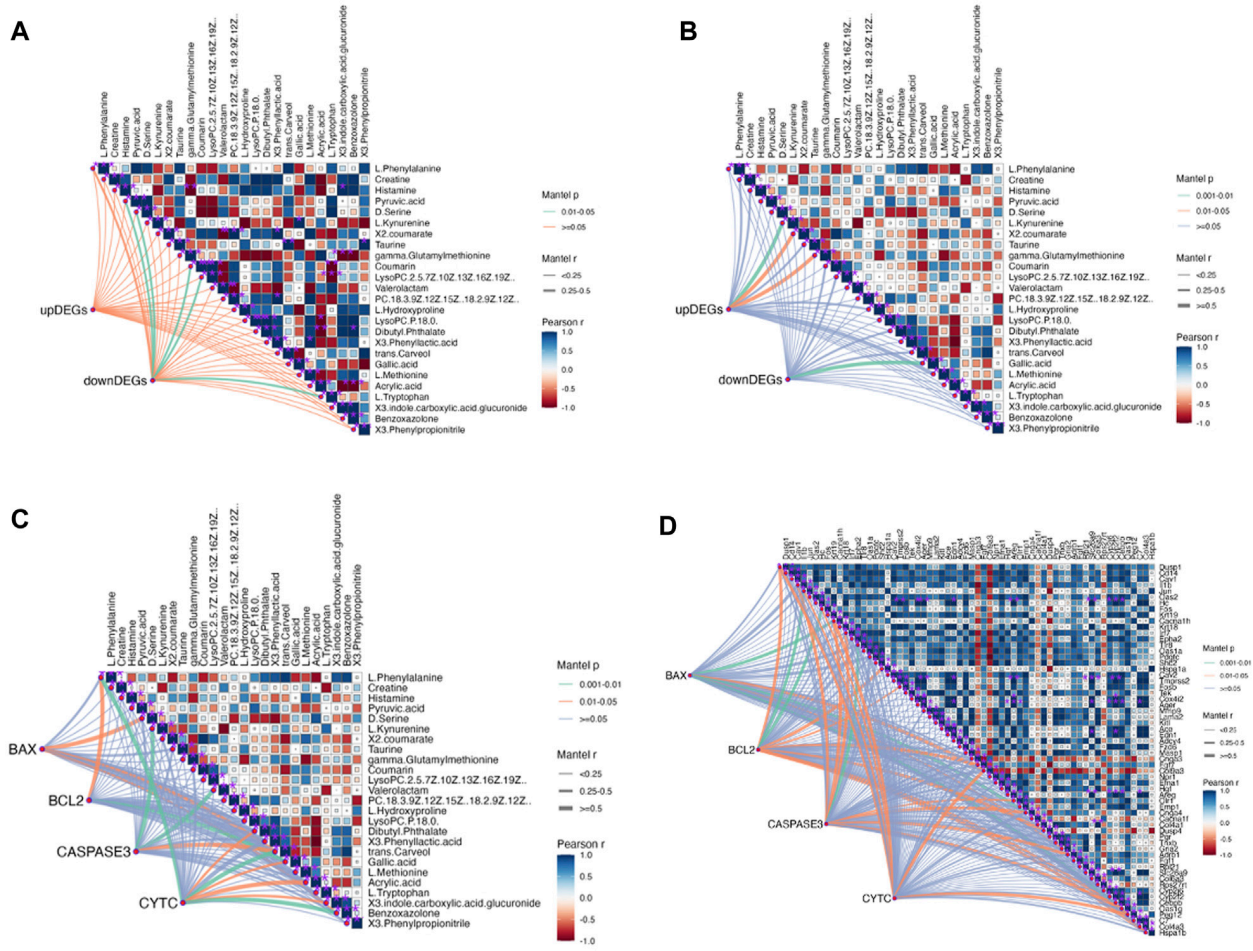
Correlation analysis between key proteins concentration, DEGs and DMs performed by Mantel test in RStudio. The orange color curve indicated significant correlation, the green color curve in-dicated statistical difference, and the purple curve indicated no significant correlation. **(A)** Correlation analysis between DMs and DEMs. **(B)** Correlation analysis between Path-way-related genes and DMs. **(C)** Correlation analysis between key proteins concentration and DMs. **(D)** Correlation analysis between key proteins concentration and Pathway-related genes.

Further analysis was conducted to explore the mechanism of QJSB in treating leukopenia, including correlation analysis of apoptotic proteins, metabolomics, and transcriptomics. The correlation analysis revealed a connection between apoptotic protein expression and amino acid metabolism (Figure 8B), particularly with phenylalanine and kynurenine. Additionally, downregulated genes were associated with lipid metabolism, such as LysoPC (22:5 (7Z,10Z,13Z,16Z,19Z)) and L-Tryptophan. Upregulated genes were correlated with D-Serine, L-Kynurenine, and Taurine (Figure 8A). Furthermore, the correlation analysis of pathway genes and proteins showed a positive relationship between JUN and FOS genes and BCL2 and CYTC proteins (Figure 8B, C), indicating that QJSB modulates apoptosis by regulating key genes like JUN and FOS.

## 4 Discussions

In 2020, cancer rates worldwide will remain high, with chemotherapy being a key treatment. However, a common side effect of chemotherapy is leukopenia, which can lead to serious infections and bleeding (Tian et al., 2019; Chen et al., 2023). QJSB, a traditional Hmong folk formula, has shown significant efficacy in treating leukopenia and granulocyte deficiency. Its potential active ingredients for treating leukopenia are abundant, as supported by experimental and clinical data (Chen et al., 2023). According to serum pharmacochemistry theory, TCM ingredients can have therapeutic effects when they enter the bloodstream (Song et al., 2023). All the components in QJSB come from natural plant sources, which have less toxic side effects than chemical drugs. Most Chinese medicines are non-toxic or have low toxicity, but they are definitely not the so-called generalized “Chinese medicines are natural medicines without any toxic side effects”. Chinese medicine recognizes diseases from the macro level, pays attention to the overall balance of treatment, and most of the drugs are plants rather than single chemical components, with low targeting, so the toxicity of Chinese medicine treatment is relatively low. To determine which compounds from QJSB enter the bloodstream, we used UHPLC-ESI-Q-Exactive Plus Orbitrap MS to analyze the *ex vivo* material basis of QJSB (Miao et al., 2018). We identified 16 prototype compounds in the serum of mice that were administered QJSB (Wang et al., 2022b; Ma et al., 2022; Ma et al., 2024). Among these compounds, Matrine was found to reverse the effects of sepsis-induced STRT1 downregulation and deacetylation of p53 and the NF-kB p65 subunit (Yang et al., 2023), thereby blocking the p53-induced apoptotic pathway in septic lung and inactivating the NF-kB pathway. Additionally, Sophoridine was found to protect brain cells in pMCAO mice by up-regulating BCL2 and down-regulating the expression levels of BAX and CASPASE3, thus preventing apoptosis. During molecular docking simulation, Daidzein, Matrine, and Sophoridine were found to bind to these apoptotic proteins with affinity energies below −5.0 kcal/mol. Meanwhile, during molecular docking simulations, the affinity energies of Daidzein, Matrine, and Sophoridine binding to these apoptotic proteins were found to be lower than −5.0 kcal/mol, which suggests, to some extent, that JUN, BCL2, CASPASE3, and FOS are the key targets of QJSB to ameliorate leukopenia.

Our research indicated that the active components in QJSB could work together to regulate cell proliferation and growth, potentially improving leukopenia (Wang et al., 2022a). We created a mouse model of CTX-induced leukopenia and observed a significant increase in peripheral leukocyte counts in CTX mice treated with QJSB, a key indicator of immune changes (Bertelsen et al., 2011). The positive drug leucogen also showed therapeutic effects compared to the model mice. The QJSB group (M) was particularly effective in increasing peripheral leukocytes, leading us to choose this dose for further experiments. Additionally, the spleen is an important immune organ in mammals, responsible for storing red blood cells and housing cells like dendritic cells, macrophages, and lymphocytes (Kaur et al., 1974; Qu et al., 2016). Our study found that QJSB increased the number and percentage of CD4^+^ T cells in the spleen of CTX mice, as well as improving the splenic index, indicating enhanced immune function in these mice (Cao et al., 2023).

In studies on metabolomics, the administration of QJSB was found to increase the levels of LysoPC (22:5 (7Z,10Z,13Z,16Z,19Z)), Histamine, and D-Serine, while decreasing the levels of L-Phenylalanine, LysoPc (P-18:0), L-Kynurenine, L-Tryptophan, and other compounds. A significant decrease in plasma lysophosphatidylcholine (LPC) was observed, which was restored after treatment with QJSB. Previous research has shown that amino acid and glycerophospholipid metabolism play a crucial role in hematopoiesis (Lecoq, 1957). Amino acids are essential for cell growth, survival, and division, and regulating amino acid metabolism has been shown to have therapeutic effects on leukopenia (Langhammer et al., 2011). Additionally, hematopoietic dysfunction has been linked to glycerophospholipid metabolism (Kinoshita and Matsumori, 2022), suggesting that QJSB may improve leukopenia by influencing these metabolic pathways.

The analysis of serum medicinal chemistry revealed that many components of QJSB are closely related to the cell growth process, leading to the hypothesis that the mechanism of QJSB in treating leukopenia may involve the regulation of cell growth. Furthermore, a “QJSB-active component-pathway” network was constructed, identifying five targets (BCL2/CASPASE3/JUN/FOS/HGF) with significant enrichment. Transcriptomics results showed changes in the expression of JUN, FOS, and HGF genes in both the model group and the QJSB group, further supporting the potential role of QJSB in regulating cell growth processes for the treatment of leukopenia. According to correlation analysis, there was a positive correlation between JUN and FOS genes and BCL2 and CYTC proteins. Differential gene changes have been identified, with these genes being common targets for multi-omics integration. They play crucial roles in cell proliferation (Vanhaesebroeck et al., 2010), differentiation (Wakeley et al., 2020), and apoptosis (Jafari et al., 2019), and are essential for maintaining homeostasis and regulating processes such as hematopoiesis (Meloche and Pouysségur, 2007), erythropoiesis, and leukocytogenesis (Liu et al., 2010; Wang et al., 2020). The cytokine HGF is involved in mediating antiapoptotic signals through AKT and plays a role in angiogenesis (Chaparro et al., 2015), neurogenesis, organogenesis, and tissue regeneration (Bu et al., 2011). Inhibiting FOS signaling may enhance the growth of hematopoietic stem cells (Wei et al., 2014) research by Liebermann et al. (1998). Has shown that FOS can promote apoptosis in hematopoietic progenitor cells of the myeloid lineage, while other studies suggest that FOS may protect against apoptosis in mammalian cells. The role of FOS in apoptosis regulation appears to be cell type and environment-dependent. JUN, a well-studied member of the AP-1 transcription factor family, is implicated in various cellular processes, including cancer, survival, apoptosis, proliferation, and tissue development (Meng and Xia, 2011).

In this study, we firstly used network pharmacology, metabolomics, and transcriptomics to investigate the mechanism of astragalus gum to enhance white capsule on leukopenia, and the results enriched to multiple pathways. The enriched pathways include PI3K-AKT signaling pathway, MAPK signaling pathway, phenylalanine metabolism, glycerophospholipid metabolism, etc., which are all closely related to the cell growth process. QJSB is a large group prescription Miao drug with a complex molecular mechanism, so we validated its key core targets by integrating network pharmacology and multi-omics pathways and targets in order to conduct an in-depth study of its complex mechanism. During the validation experiments, we simulated the binding of blood-entry components and key proteins through molecular docking, and the results showed that their binding energies were all below −5.0 kcal/mol; secondly, we found that QJSB could inhibit the expression of pro-apoptotic proteins and inhibit apoptosis through Western Blot; Therefore, we proposed that QJSB could improve leukopenia by inhibiting T cell apoptosis. It is suggested that QJSB may modulate immune and hematopoietic processes by targeting these genes, influencing cell proliferation and apoptosis, and potentially improving leukopenia induced by CTX and increasing leukocyte levels.

**FIGURE 9:**
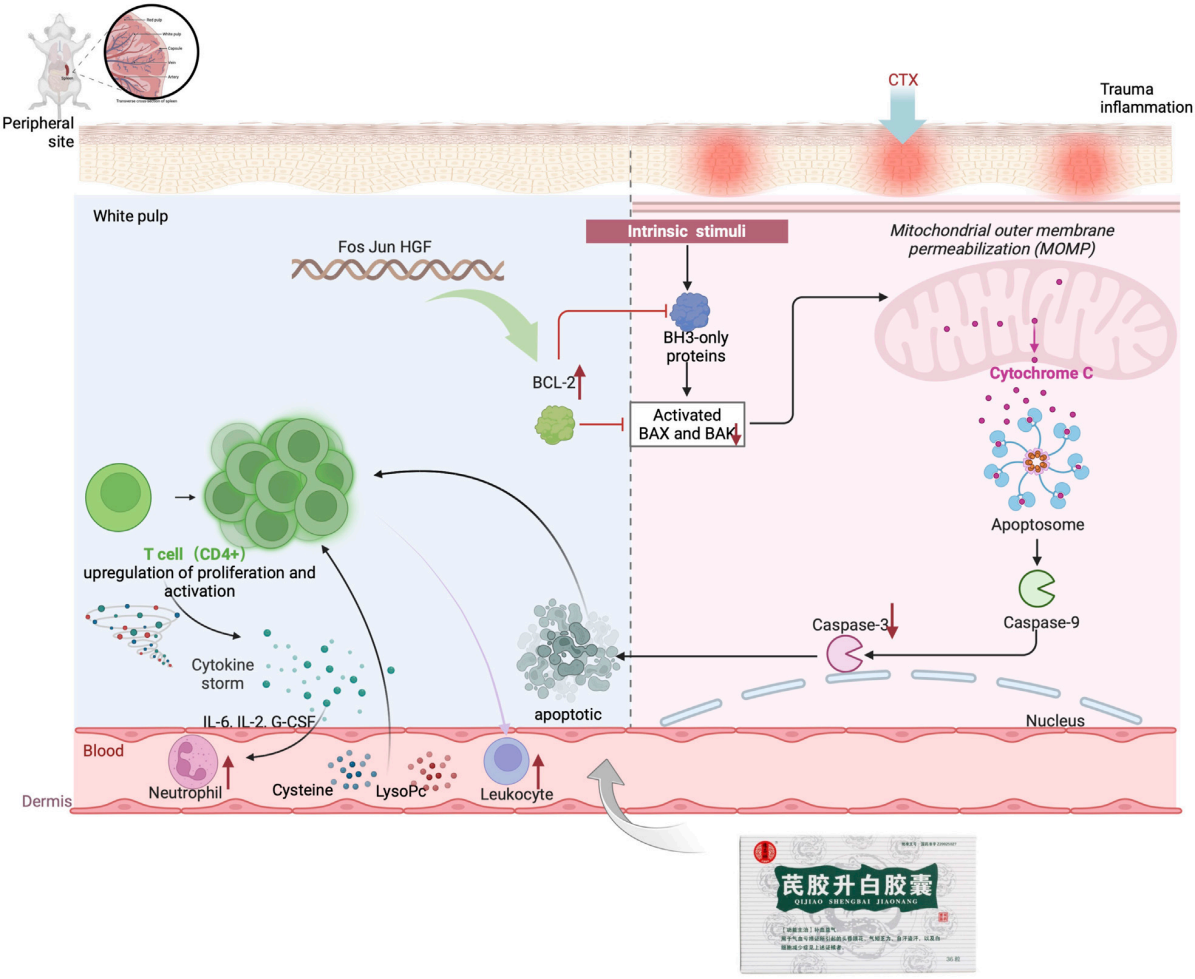
QJSB improvement mechanism of leukopenia diagram.

The mitochondrial apoptotic pathway is typically activated in response to cellular stress or injury, and is regulated by interactions between members of the Caspase and BCL2 protein families (Bock and Tait, 2020). Under normal conditions, BAX binds to BCL2 proteins to prevent cell death. However, in the presence of toxic substances, BAX can increase the permeability of the mitochondrial membrane, leading to the release of apoptosis-inducing substances (Sahu et al., 2014; Peña-Blanco and García-Sáez, 2018). In this study, it was observed that the Model group had increased BAX expression and decreased BCL2 expression, while the QJSB group showed the opposite effect. This suggests that QJSB may inhibit Bax activation and promote BCL2 expression, thereby preserving mitochondrial integrity. Furthermore, the imbalance between Bax and BCL2 can lead to the release of Cytochrome C (CYTC), which triggers a cascade of events culminating in the activation of Caspase-3 and subsequent cell death (Dorstyn et al., 2018). The QJSB group exhibited lower levels of CYTC expression and reduced Caspase-3 activation compared to the CTX-treated group, indicating that QJSB may effectively regulate the mitochondrial pathway and prevent cell death in CD4^+^ T lymphocytes, ultimately improving leukopenia. Although it has been reported that QJSB may have anti-inflammatory properties, interfere with the leukotriene pathway, and improve hematopoiesis in leukopenic mice (Ma et al., 2024), our results show that QJSB can also improve leukopenia by increasing T cell count, reversing splenic damage, enhancing splenic immune function, and reducing apoptosis by inhibiting apoptotic protein expression. This is because the herbal compound acts through multiple targets, components, and pathways, providing strong evidence for its effectiveness. Figure 9.

## 5 Conclusion

To sum up, we created a mouse model of CTX-induced leukopenia. Based on this, we screened important targets and signaling pathways by integrating transcriptomics, metabolomics, and network pharmacology analyses. Through *in vivo* investigations, the effects of QJSB on peripheral and splenic serum metabolites and their processes were examined. The outcomes demonstrated that QJSB ameliorates leukopenia by affecting the expression of apoptotic proteins associated with the mitochondrial pathway. It was revealed in the results that QJSB had the ability to enhance T cell numbers and reduce the expression of apoptotic proteins, leading to improved immune function and an increase in leukocyte count.

## Data Availability

The datasets presented in this study can be found in online repositories. The names of the repository/repositories and accession number(s) can be found below: https://www.ebi.ac.uk/metabolights/MTBLS10528, accession number: MTBLS10528; https://www.ebi.ac.uk/metabolights/MTBLS10945, accession number: MTBLS10945 https://www.ncbi.nlm.nih.gov/, accession number: PRJNA1127432.
