# Supplementary material for: Mechanistic study of leukopenia treatment by Qijiao shengbai Capsule via the Bcl2/Bax/CASAPSE3 pathway
Source: Front Pharmacol. 2024 Sep 4;15:1451553. doi: 10.3389/fphar.2024.1451553 (PMC11408280; doi:10.3389/fphar.2024.1451553)
Supplement: Supplementary file 1 [file DataSheet1.docx]

Mechanistic study of leukopenia treatment by Qi Jiao Sheng Bai capsule via the Bcl2/Bax/Casapse3 pathway

Siyue Jiang*, Pengjiao Wang *, Xiaodong Sun, Min Zhang, Shuo Zhang, Yu Cao, Yuben Wang

*** Correspondence:** Xiuli Gao: gaoxl@gmc.edu.cn and Li Liu: 382041322@qq.com

**Fig.S1**

**
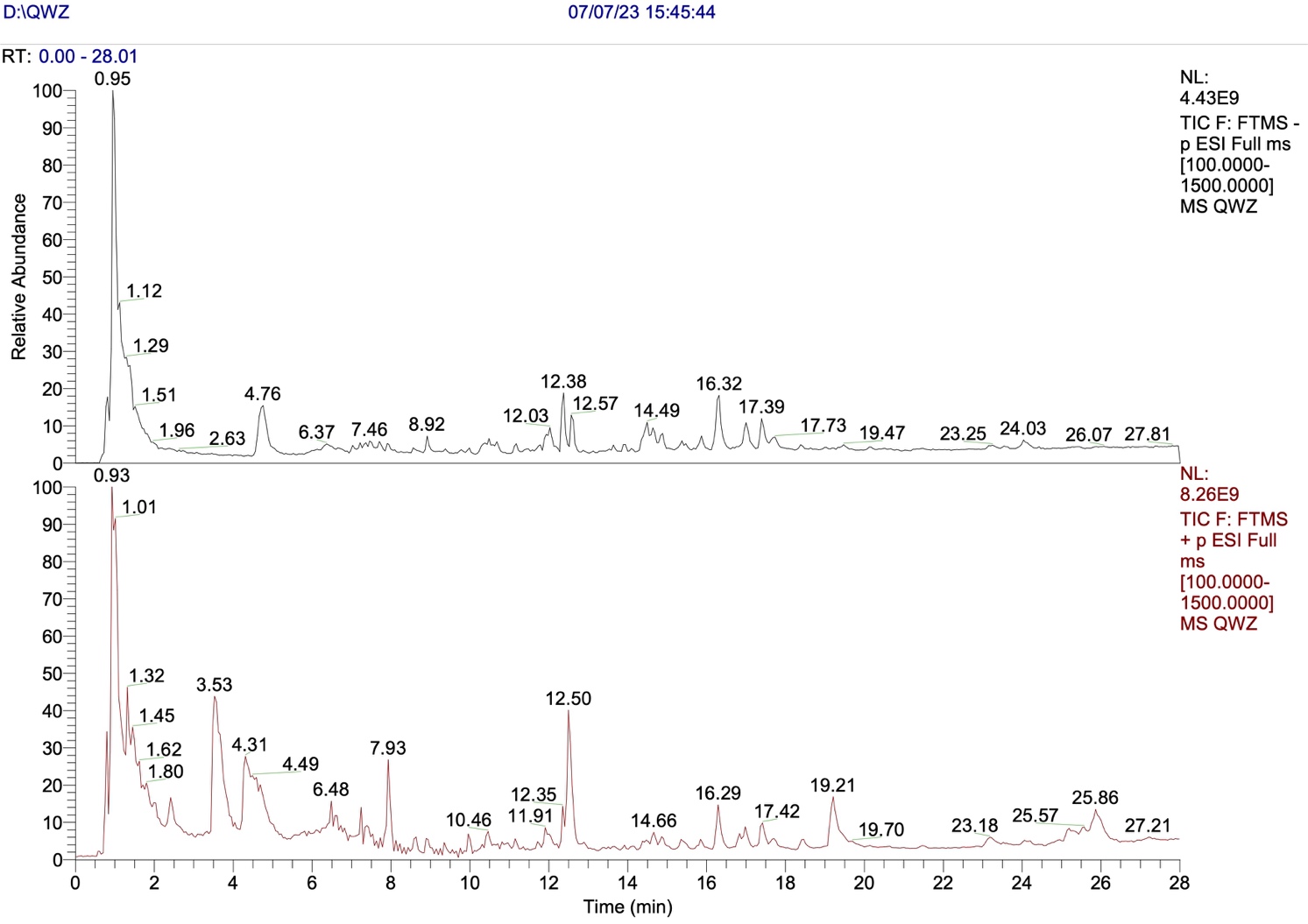
**

**Fig.S1.** Total ion chromatogram of QJSB

**Fig.S2**


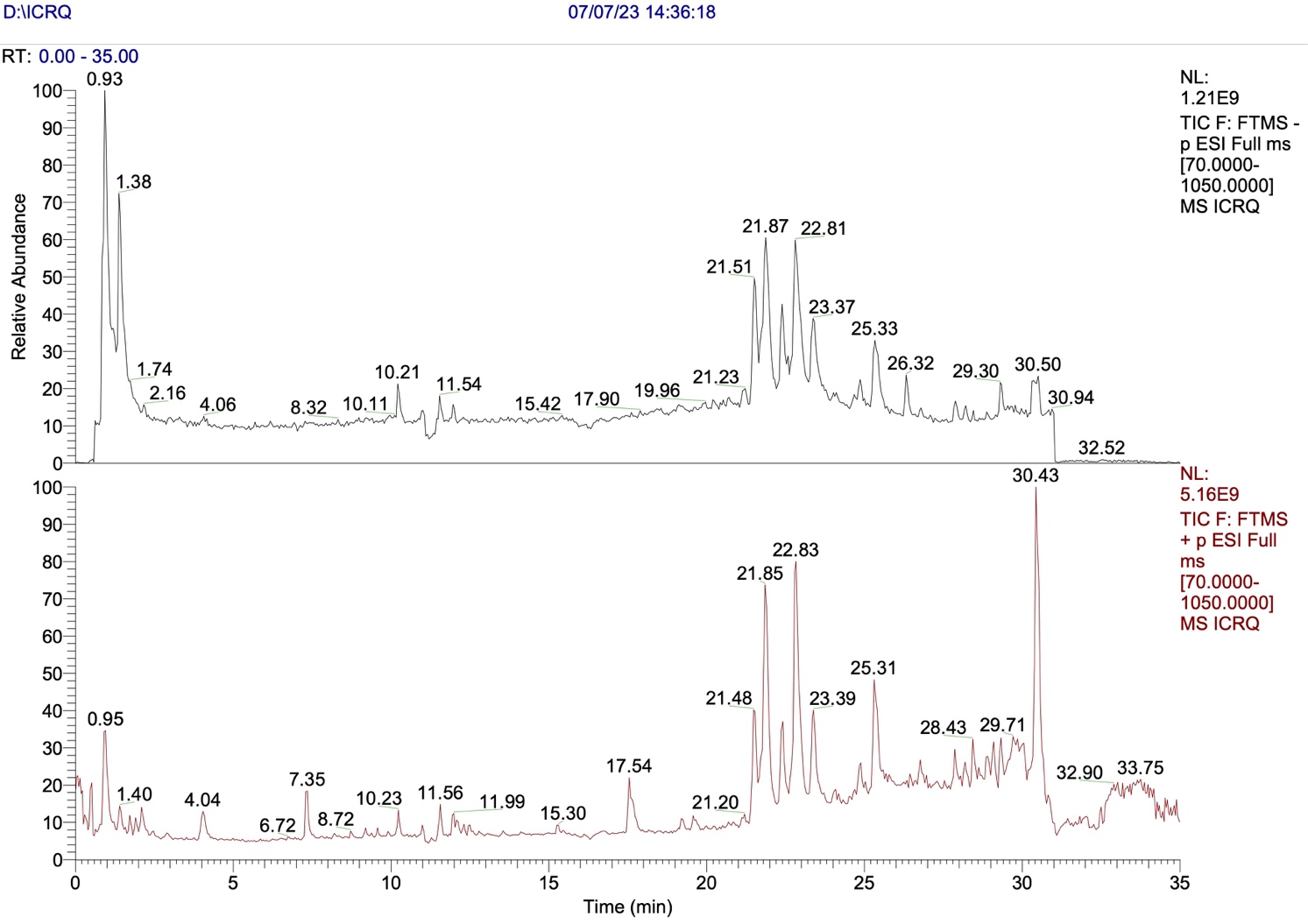


**Fig.S2.** Total ion chromatogram of serum pharmacochemical samples

**Fig.S3**


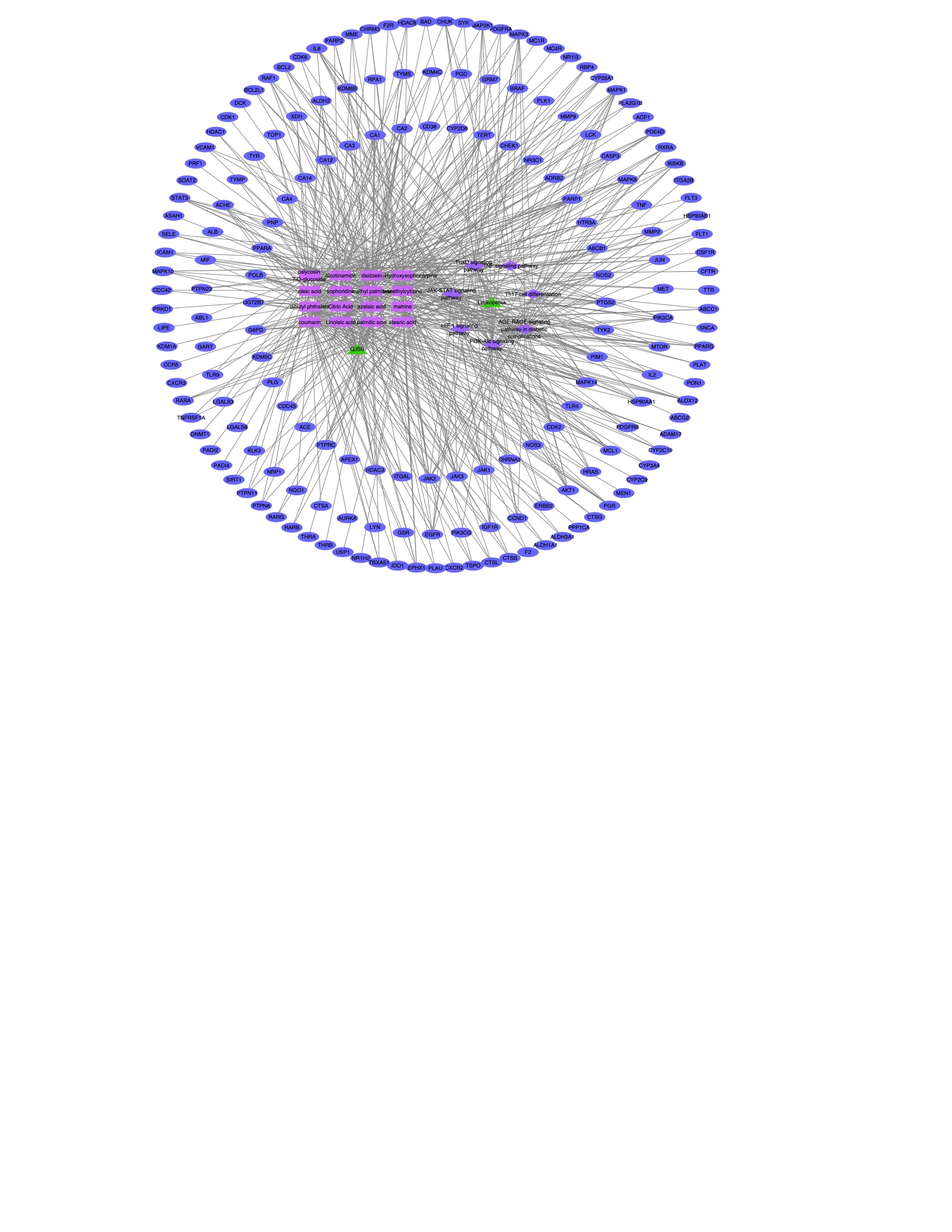


**Fig.S3.** A blood entry component-target-pathway diagram
